# Engineering Active PET Packaging via Corona Treatment and Natural Biocide Coating: Carvacrol and *Trans*-Cinnamaldehyde for Food Preservation

**DOI:** 10.3390/polym18070809

**Published:** 2026-03-26

**Authors:** Pantelis Karaboulis, Areti A. Leontiou, Christos Tsakonas, George Paterakis, Margarita Dormousoglou, Andreas Giannakas, Panagiota Stathopoulou, Charalampos Proestos, Costas Galiotis, Constantinos E. Salmas, Aris E. Giannakas

**Affiliations:** 1 Department of Food Science and Technology, University of Patras, 30100 Agrinio, Greece; up1120174@upatras.gr (P.K.); aleontiu@upatras.gr (A.A.L.); andgiannakas@upatras.gr (A.G.); 2 Department of Chemical Engineering, University of Patras, 26504 Patras, Greece; ctsako@chemeng.upatras.gr (C.T.); gpaterakis@iceht.forth.gr (G.P.); c.galiotis@iceht.forth.gr (C.G.); 3 Institute of Chemical Engineering Sciences, Foundation for Research and Technology—Hellas (FORTH/ICE-HT), 26504 Patras, Greece; 4 Department of Sustainable Agriculture, University of Patras, 30100 Agrinio, Greece; m.dormousoglou@upatras.gr (M.D.); panstath@upatras.gr (P.S.); 5 Laboratory of Food Chemistry, Department of Chemistry, National and Kapodistrian University of Athens, 15771 Athens, Greece; harpro@chem.uoa.gr; 6 Department of Material Science and Engineering, University of Ioannina, 45110 Ioannina, Greece

**Keywords:** corona treatment, active packaging, essential oils, nanocoating, carvacrol, *trans*-cinnamaldehyde, natural biocide, food preservation, minced meat, table olives

## Abstract

**Featured Application:**

This work presents a laboratory-scale proof-of-concept demonstrating the potential to transform conventional PET packaging into active food preservation containers by combining corona discharge treatment—a process already ubiquitous in the packaging industry—with natural biocide coatings of carvacrol and trans-cinnamaldehyde. The approach demonstrates feasibility for further development toward industrial application. The PET-*t*CN system demonstrates exceptional potential for extending refrigerated shelf-life of fresh meat by 3–4 days and preserving table olives under ambient storage for up to 21 days, while reducing oxygen transmission by 80%. The technology leverages existing industrial infrastructure, preserves PET’s mechanical integrity, transparency, and recyclability, and offers food producers a readily adoptable solution for enhancing food safety, reducing waste, and meeting consumer demand for clean-label, naturally preserved products.

**Abstract:**

The food packaging industry requires sustainable solutions to reduce plastic waste and replace synthetic additives. This study addresses the need for scalable methods to transform conventional polyethylene terephthalate (PET) packaging into active food preservation systems using natural biocides. Commercial PET packaging was surface-activated using industrial-scale corona treatment, followed by coating with natural biocides—carvacrol (CV) and *trans*-cinnamaldehyde (*t*CN). The resulting active packaging materials (PET-CV and PET-*t*CN) were characterized using XRD, FTIR, SEM, AFM, and desorption kinetics. Packaging properties including mechanical strength, oxygen barrier, antioxidant (DPPH), and antibacterial activity (against *S. aureus* and *E. coli*) were evaluated. Real-food preservation tests were conducted using fresh minced pork (4 °C, 6 days) and table olives (23 °C, 21 days), monitoring microbiological (TVC), colorimetric (CIE L*a*b*), and pH changes. Corona treatment successfully anchored both biocides through physical adsorption, with *t*CN exhibiting stronger surface interaction (desorption energy: 128.0 kJ/mol). Both coatings significantly improved oxygen barrier properties (61% reduction for PET-CV, 80% for PET-*t*CN). PET-*t*CN demonstrated superior antibacterial activity (inhibition zones: 15.0 mm against *E. coli*). In pork preservation, PET-*t*CN achieved a 2-log reduction in TVC, maintained meat redness (a*: 12.80 vs. 5.10 for control), and stabilized pH. For olives, PET-*t*CN reduced TVC by 2.35 log cycles and preserved green color. This corona-assisted coating approach, demonstrated here at laboratory scale, successfully transforms inert PET into multi-functional active packaging with potent antimicrobial, antioxidant, and barrier properties, significantly extending food shelf-life and offering a sustainable solution for reducing food waste.

## 1. Introduction

In the context of a global shift towards a sustainable circular economy, the food packaging industry faces mounting pressure to reduce reliance on non-renewable resources and synthetic chemical additives [1,2,3,4,5,6,7,8]. A key strategy involves the development of advanced, functional materials that extend food shelf life while minimizing environmental impact [5,6,9,10,11,12]. This has spurred significant interest in replacing conventional antioxidant and antimicrobial agents with natural, biobased alternatives [13,14,15]. Among these, plant-derived essential oils (EOs) stand out as potent sources of bioactive compounds, such as phenolics and terpenes, known for their broad-spectrum antimicrobial and antioxidant activities [16,17]. Integrating these natural biocides into packaging materials to create “active packaging” represents a promising avenue for enhancing food preservation, reducing food waste, and meeting consumer demand for clean-label products [18,19,20,21].

Two of the most widely studied EO components for active food packaging are carvacrol (CV) and trans-cinnamaldehyde (*t*CN). CV, a monoterpenoid phenol abundant in oregano and thyme oils, exhibits strong antibacterial activity against both Gram-positive and Gram-negative bacteria, including foodborne pathogens such as *Listeria monocytogenes* and *Escherichia coli*, primarily through the disruption of microbial cell membranes [22,23]. Similarly, trans-cinnamaldehyde, the principal aldehyde in cinnamon bark oil, possesses broad-spectrum antimicrobial and antifungal properties, alongside notable antioxidant capacity, making it effective against spoilage microorganisms and oxidative rancidity in foods [24,25,26,27]. Their volatility and hydrophobicity, however, pose challenges for direct incorporation into polymers, often requiring encapsulation or chemical modification to achieve controlled release and maintain activity [28,29,30,31,32]. Successfully anchoring these potent but challenging compounds onto packaging surfaces remains a key technological hurdle.

Polyethylene terephthalate (PET) has emerged as one of the dominant materials in the food packaging sector due to its excellent combination of mechanical strength, optical clarity, and barrier properties against gases and moisture [33]. Recent comprehensive reviews have highlighted both the opportunities and challenges associated with PET in food packaging applications, including issues related to recycling, sustainability, and the need for functional modifications to extend shelf life [33]. The versatility of PET has led to its widespread adoption for bottles, containers, and films, yet its inherent surface inertness limits its direct use in active packaging systems without surface modification [34]. However, its inert and low-surface-energy nature presents a significant challenge for the direct and stable application of functional coatings [35]. To overcome this, surface activation techniques are crucial. Critically, this study leverages corona discharge treatment—a process already ubiquitously employed in the printing and packaging sectors to improve ink adhesion on polymer surfaces [36,37]—here applied at laboratory scale to demonstrate the feasibility of the approach. This established, cost-effective, and scalable technology modifies polymer surfaces by introducing polar functional groups (e.g., carbonyls, hydroxyls) through atmospheric plasma oxidation, thereby increasing surface energy and wettability to enhance the adhesion and uniformity of subsequently applied coatings [38].

Recent research has explored the incorporation of various EOs, including CV and cinnamaldehyde, into biopolymer or synthetic polymer matrices [39,40]. However, the direct and efficient grafting of these EO components specifically onto the inner surface of pre-formed, commercial PET packaging, utilizing this industrially mature corona process as the key activation step, remains underexplored. Many studies focus on blend films or complex multilayers, which may compromise transparency, scalability, or require extensive material reformulation [41,42]. There is a distinct need for a scalable methodology that transforms conventional PET packaging into an active system without altering its bulk properties or complicating its recyclability stream, ideally using technologies already embedded in manufacturing lines.

This study introduces an innovative, two-step approach to engineer active PET packaging by synergizing established industrial practice with natural chemistry: (1) the industrial-compatible corona treatment of the package’s interior surface to activate it, followed by (2) the direct coating with two potent natural biocides—CV and trans-cinnamaldehyde. The innovation lies in creating a stable, surface-bound active layer on commercially available PET by repurposing a standard industrial process (corona) for a novel functional outcome (EO anchoring). The developed materials were comprehensively characterized using X-ray diffraction (XRD), Fourier-transform infrared spectroscopy (FTIR), scanning electron microscopy (SEM), atomic force microscopy (AFM), and desorption kinetics analysis to confirm coating success, morphology, and controlled release behavior. Furthermore, the active packages were evaluated for their tensile strength, oxygen barrier properties, and in vitro antioxidant and antibacterial efficacy. Finally, the practical application and shelf-life extension capability of this novel active packaging were demonstrated through real-food preservation tests using highly perishable fresh pork mince and table olives as model systems. This work provides a viable pathway for upcycling standard PET packaging into high-value, active food-preserving containers, contributing to both food safety and sustainability goals. The key contribution of this work is the successful laboratory-scale demonstration of a process to impart strong and functional antibacterial/antioxidant activity to the interior of commercial PET packaging by repurposing corona treatment—a method widely used in industry—for the stable anchoring of natural essential oil coatings, creating a fully characterized active material with proven efficacy in real-food systems. This proof-of-concept study establishes the feasibility of the approach, which has potential for industrial scalability pending further development and validation.

## 2. Materials and Methods

### 2.1. Materials

Commercial semi-transparent PET packaging containers were supplied by the local market (Praktika, Agrinio, Greece). Carvacrol (CV) (CAS: 499-75-2), *trans*-cinnamaldehyde (*t*CN) (CAS:14371-10-9), and 2,2-diphenyl-1-picrylhydrazyl (DPPH) (CAS:1898-66-4) were obtained from Sigma-Aldrich (Darmstadt, Hesse, Germany).

### 2.2. Surface Coating of PET Packaging with CV and tCN

The inner surfaces (both top and bottom) of the PET packaging were coated via a handheld corona surface treatment. A BD-20 V model (Electro-Technic Products, Chicago, IL, USA) was used, with an output voltage range of 10–45 kV and an output power range of 5–30 W.

Carvacrol (CV, purity ≥98%) and trans-cinnamaldehyde (*t*CN, purity ≥ 99%) were obtained from Sigma-Aldrich (Darmstadt, Hesse, Germany) and were used as received, without any solvent or dilution. Immediately following corona treatment, a precise volume of 100 µL of the pure biocide was applied per 25 cm^2^ of the activated PET surface. The biocide was spread uniformly using a clean, fine-bristle paintbrush for approximately 30 s to ensure a continuous, thin liquid layer. The coated packages were then allowed to stabilize in a fume hood at ambient temperature (23 ± 2 °C) for 24 h to allow for stable surface interaction and the evaporation of any volatile components. The resulting surface-coated packages are hereafter referred to as PET-CV and PET-*t*CN.

### 2.3. Physicochemical Characterization of Surface Coated PET-CV and PET-tCN

#### 2.3.1. XRD Analysis

The obtained surface coated PET-CV and PET-*t*CN packages as well as the pure PET package were characterized with XRD analysis by using a Bruker D8 Advance diffractometer (Billerica, MA, USA) with Cu-Kαradiation (λ = 1.5406 Å) in the 2θ range of 5–40°.

#### 2.3.2. ATR-FTIR Spectrometry

ATR-FTIR spectra of pure PET, corona-treated PET (PET-co), as well as surface coated PET-CV and PET-*t*CN samples, were recorded on a Shimadzu FT-IRSpirit spectrometer (Kyoto, Japan) equipped with an ATR accessory, over the range of 4000–400 cm^−1^ at a resolution of 4 cm^−1^. ATR-FTIR spectrometry was also used as a key method to confirm the surface bonding of CV and *t*CN molecules on PET. For this reason, surface-coated PET-CV and PET-*t*CN samples were prepared and keep in desiccator for 2 months under ambient conditions. Their ATR-FTIR spectra was measured on day 0, day 10, day 30 (1 month), and day 60 (2 months).

#### 2.3.3. CV and tCN Desorption Kinetics from Surface Coated PET-CV and PET-tCN

The desorbed amounts of CV and *t*CN from the NZ surface, along with their release rates, were determined through desorption kinetic experiments conducted on both surface-coated PET-CV and PET-*t*CN samples.

Approximately 100 mg of each PET-CV and PET-*t*CN sample was placed in a AXIS AS-60 (AXIS Sp. z o.o. ul. Kartuska 375b, 80–125 Gdansk, Poland), moisture analyzer. The change in mass was recorded continuously over time at three set temperatures: 50, 60, and 70 °C. All measurements were conducted in triplicate to confirm consistency.

From the recorded mass over time ($m_{t}$), a normalized released fraction, $q_{t}$, was computed for each dataset using the expression:

(1) $$q_{t}=1-\frac{m_{t}}{m_{0}}$$

where $m_{0}$ denotes the initial mass of the sample. The calculated $q_{t}$ values were then graphed as a function of time for analysis at each temperature.

The resulting release profiles ($q_{t}$ vs. time) were analyzed by fitting them to a pseudo-second-order kinetic model. The governing differential equation for this model is:

(2) $$\frac{dq_{t}}{dt}=k_{2}(q_{e}-q_{t}{)}^{2}$$

In this equation, $k_{2}$ represents the model’s rate constant (s^−1^), $q_{t}$ is the fraction released at time t, and $q_{e}$ is the estimated fraction released at equilibrium.

The integrated form of this model, used for the fitting process, is given by:

(3) $$q_{t}=\frac{q_{e}^{2}k_{2}t}{q_{e}k_{2}t+1}$$

The curve-fitting analysis yielded the best-fit values for the kinetic parameters $k_{2}$ and $q_{e}$ at every tested temperature. From these parameters, the initial release rate ($r_{i}$) was determined using the formula:

(4) $$r_{i}=k_{2}\cdot q_{e}^{2}\;(4)$$

To estimate the activation energy associated with the mass loss process ($E_{des}^{0}$), an Arrhenius relationship was applied. The natural logarithm of the rate constants ($\ln k_{2}$) was plotted against the inverse absolute temperature (1/T). This relationship is described by the linear equation:

(5) $$\ln\left(k_{2}\right)=\ln\left(k_{0}\right)-\frac{E_{des}^{0}}{RT}$$

where $k_{0}$ is the frequency factor and R is the gas constant. The slope of this linear plot provided the value for the activation energy ($E_{des}^{0}$), following the established theoretical framework cited in the literature.

#### 2.3.4. SEM Images

Surface coated PET-CV and PET-tCN were examined using a scanning electron microscope (SEM) (FEI Quanta 250 FEG (FEI Company, Hillsboro, OR, USA)) working under different pressures (10–4000 Pa).

#### 2.3.5. AFM Images

Surface coated PET-CV and PET-tCN were collected using Dimension Icon microscope (Bruker) operating in Peak Force Tapping mode using RTESPA-300 probes (nominal spring constant 40 N/m, frequency ∼300 kHz). No data treatment apart from line subtraction (retrace) to remove the tilt has been performed.

#### 2.3.6. Surface Energy Measurement

The surface energy of the PET films was determined using ACCU DYNE TEST™ Marker Pens (ACCU Dyne Test, Diversified Enterprises, Claremont, NH, USA), following the manufacturer’s standard procedure and the principles of ASTM D2578. A set of pens with gradually increasing dyne levels (from 30 to 72 dynes/cm) were employed. For each measurement, a line was drawn with a pen across a flat, smooth area of the sample. The surface energy (in dynes/cm) was recorded as the highest dyne-level pen whose fluid formed a continuous film on the surface for at least 2 s without beading. Measurements were performed in quintuplicate (*n* = 5) on different areas of each sample type: unmodified PET, corona-treated PET (PET-co), PET-CV, and PET-*t*CN. The results are reported as the mean value with the observed range.

#### 2.3.7. Determination of Coating Thickness

The thickness of the CV and *t*CN coatings was estimated using a gravimetric method on circular substrates. Five circular PET coupons with a diameter of 3.8 cm (radius = 1.9 cm) were weighed individually. They were then corona-treated and coated with the biocide following the procedure in Section 2.2. After the 24 h stabilization period, the coated coupons were weighed again. The mass of the applied coating was calculated by subtracting the initial mass from the final mass. The coating thickness (h) was estimated using the formula derived from the geometric relationship: mass = density × volume, and for a thin coating on a flat substrate, volume = area × height. Thus, h = m/(ρ × A), where m is the average mass of the coating (g), ρ is the density of the biocide (CV: 0.977 g/cm^3^; *t*CN: 1.05 g/cm^3^), and A is the coated surface area (A = πr^2^ = π × (1.9 cm)^2^ ≈ 11.34 cm^2^). The calculated parameters and final thickness are presented in Section 3.1.7. The thickness is reported in micrometers (µm) as mean ± standard deviation.

#### 2.3.8. Optical Transparency

The optical transparency of unmodified PET, PET-CV, and PET-*t*CN films was evaluated using UV-Vis spectroscopy. Film specimens (1 cm × 4 cm) were cut from the bottom surface of the packages and placed directly in the beam path of a SHIMADJU UV/VIS-1900 (Shimadzü, Kyoto, Japan) spectrophotometer. Transmittance spectra were recorded over a wavelength range of 400–700 nm, corresponding to the visible light spectrum. Air was used as the blank. Measurements were performed in triplicate for each sample type.

### 2.4. Packaging Properties Characterization of Surface Coated PET-CV and PET-tCN

#### 2.4.1. Tensile Properties

The mechanical tensile properties—specifically Young’s modulus, tensile strength, and elongation at break—were characterized following the standard test method ASTM D638. Specimens of standard dimensions (typically Type I or Type IV) were prepared from the polymer films and conditioned prior to testing. Measurements were performed using a Shimadzu AG-X plus universal testing machine (Kyoto, Japan). Each sample was mounted in the machine’s grips and subjected to a controlled, constant-rate extension (10 mm/min) until failure. A minimum of five specimens were tested for each sample type to ensure statistical reliability. The Young’s modulus was calculated from the initial linear slope of the stress–strain curve, the tensile strength was determined as the maximum stress before failure, and the elongation at break was recorded as the strain at the point of rupture.

#### 2.4.2. Oxygen Barrier Properties

The oxygen barrier performance of the films was evaluated by measuring the Oxygen Transmission Rate (OTR) in accordance with ASTM D3985. Tests were conducted using an Oxygen Permeation Analyzer (Model 8001, Systech Illinois, Johnsburg, IL, USA). For each measurement, a preconditioned film sample was securely mounted in a test cell, creating a barrier between two chambers: one flushed with an oxygen gas stream (test gas) and the other with a nitrogen carrier gas. The instrument precisely measures the amount of oxygen gas that permeates through the film over a 24 h period at a controlled temperature (23 °C) and relative humidity (0% RH, dry conditions) to determine the steady-state OTR, expressed in units of cm^3^/(m^2^·day·atm). Subsequently, the Oxygen Permeability (PeO_2_) was calculated by multiplying the obtained OTR value by the average measured thickness of the film, thereby normalizing the barrier property to the material’s intrinsic characteristic, independent of sample thickness.

#### 2.4.3. Evaluation of Antioxidant Activity (DPPH Radical Scavenging Assay)

The in vitro antioxidant capacity of the prepared nanohybrid materials and composite films was quantitatively assessed using the stable 2,2-diphenyl-1-picrylhydrazyl (DPPH) free radical scavenging assay.

Precisely weighed amounts of each sample, ranging from 5 to 50 mg, were placed in separate test tubes to establish a dose–response relationship.

To each sample, 3 mL of a freshly prepared DPPH ethanolic solution (concentration: 2.16 mM) was added. The mixtures were then incubated for 2 h at room temperature under dark conditions to prevent photo-degradation of the radical. A negative control, consisting of 3 mL of the DPPH solution without any sample, was prepared and processed under identical conditions.

After the incubation period, the reaction mixtures were centrifuged or allowed to settle to remove any undissolved particulate matter that could interfere with the spectrophotometric reading. The absorbance of the resulting supernatant was measured at a wavelength of 517 nm using a SHIMADJU UV/VIS-1900 (Shimadzü, Kyoto, Japan) spectrophotometer.

The DPPH radical scavenging activity, expressed as a percentage, was calculated for each sample concentration using the following formula:

(6) $$\%\;\mathrm{Scavenging}\;\mathrm{Activity}=\left(\frac{A_{0}-A_{s}}{A_{0}}\right)\times 100$$

where $A_{0}$ is the absorbance of the negative control (pure DPPH solution) and $A_{s}$ is the absorbance of the sample-containing DPPH solution.

The scavenging percentage was plotted against the corresponding sample concentration (mg/mL) to generate a dose–response curve. From this curve, the effective concentration (EC_50_) value—defined as the sample concentration required to scavenge 50% of the DPPH radicals—was determined by interpolation. These dose–response curves for all tested nanohybrids and films are provided in the Supplementary Materials for direct comparison.

#### 2.4.4. Evaluation of Antimicrobial Efficacy via Disk Diffusion Assay

The antibacterial performance of the surface-modified films (PET-Blank, PET-CV and PET-*t*CN) was evaluated against Gram-negative *Escherichia coli* (DSM 1103) and Gram-positive *Staphylococcus aureus* (DSM 113533), using a modified Kirby–Bauer disk diffusion method [43]. Bacteria from frozen glycerol stock cultures (stored at −80 °C) were revived and cultured on Mueller–Hinton (MH) agar plates. Before the experimental procedure, fresh isolated colonies were harvested and resuspended in sterile Phosphate-Buffered Saline (PBS). The turbidity of the suspension was adjusted to match a 0.5 McFarland standard, corresponding to a cell density of approximately 1.5 × 108 CFU/mL.

The film samples were cut into square coupons of 1 cm × 1 cm. Prior to undergoing analysis, all specimens were sterilized by irradiation with UV light on both sides to eliminate potential contamination and avoid false positives. For the inoculation, sterile cotton swabs were dipped into the standardized bacterial suspension and streaked onto the surface of MH agar plates. Subsequently, the sterilized film samples were aseptically placed onto the inoculated agar surface. The plates were incubated at 37 °C for 24 h. Following incubation, the antimicrobial capacity was quantified by measuring the diameter of the inhibition halo surrounding the samples. The final zone of inhibition was calculated by subtracting the specimen width (1 cm) from the total diameter of the clear zone. All experiments were performed in triplicate to ensure reproducibility.

#### 2.4.5. Total Viable Count (TVC) Enumeration

The total viable count (TVC) of microorganisms in food samples was determined using the standard plate count method. Food samples (10 g) were aseptically transferred to sterile stomacher bags containing 90 mL of sterile peptone water (0.1% *w*/*v*) and homogenized for 2 min. Serial ten-fold dilutions were prepared in sterile peptone water. Aliquots of 0.1 mL from appropriate dilutions were spread-plated in duplicate on Plate Count Agar (PCA, Merck, Darmstadt, Germany). Plates were incubated at 30 °C for 48–72 h. After incubation, colonies were counted, and results were expressed as log_10_ colony-forming units per gram (log CFU/g) for pork or per olive (log CFU/olive). All analyses were performed in triplicate for each sample at each time point.

### 2.5. Packaging Preservation Tests

#### 2.5.1. Preservation Test on Fresh Minced Pork

In the first preservation test, the efficacy of the surface-coated PET-based packaging systems (PET-CV, PET-*t*CN) was evaluated against unmodified PET packaging for preserving fresh minced pork, with a focus on microbiological stability, color retention, and pH changes. Fresh minced pork was obtained from a local processing facility (Ayfantis S.A., Agrinio, Greece) and transported under refrigerated conditions to the laboratory for immediate processing, where approximately 100 g portions were assigned to one of three packaging treatments: standard unmodified PET packages (PET), corona-treated PET packages with an inner surface coating of carvacrol (PET-CV), and corona-treated PET packages with an inner surface coating of trans-cinnamaldehyde (PET-*t*CN). The meat portions were sealed within their respective prepared packages and all samples were stored in a refrigerated environment maintained at a constant temperature of 4 ± 1 °C. Microbiological analysis was conducted by determining the Total Viable Count (TVC) after 2, 4, and 6 days of storage, following the method described in Section 2.4.5. Concurrently, the physicochemical quality of the meat was assessed. Surface color was measured using a portable spectrophotometer (JCS 200, KERN & SOHN GmbH, Balingen, Germany), equipped with an 8 mm measuring aperture and a D/8° viewing geometry. Color parameters were recorded in the CIE L*a*b* color space, where L* represents lightness, a* represents redness/greenness, and b* represents yellowness/blueness. The pH of the meat samples was determined using a food-grade pH meter (MW 102-FOOD PRO+, Milwaukee Instruments), equipped with a penetration probe suitable for semi-solid foods, following the manufacturer’s instructions. All color and pH measurements were performed in quintuplicate at each time point to assess and compare the preservation performance of each packaging system over the refrigerated shelf-life period.

#### 2.5.2. Preservation Test on Table Olives

In the second preservation test, the efficacy of surface-coated PET-based packaging systems (PET-CV, PET-*t*CN) was evaluated for ready-to-eat table olives, compared to standard PET packaging, under ambient storage conditions. Commercially prepared, pitted table olives were aseptically removed from their original brine, rinsed with sterile distilled water, and superficially dried. For each packaging type—standard PET, PET-CV, and PET-*t*CN—five olives were placed inside the respective package, which was then hermetically sealed. For each of the three packaging systems, nine independent replicate packages were prepared. All samples were stored in the dark at a controlled ambient temperature of 23 ± 2 °C for a total period of 21 days (3 weeks). Microbiological analysis focused solely on the Total Viable Count (TVC), conducted at weekly intervals as described in Section 2.4.5. Destructive sampling was performed on three randomly selected replicate packages from each group at each time point: Day 0 (initial), Day 7, Day 14, and Day 21. For analysis, the olives from each sampled package were homogenized in a sterile diluent for TVC determination. In parallel, the physicochemical quality of the olives was assessed. Surface color was measured directly on the olive epidermis using the same portable spectrophotometer (JCS 200, KERN & SOHN GmbH, Germany) under the CIE L*a*b* color space. The pH of the olive homogenate was measured using the food-grade pH meter (MW 102-FOOD PRO+, Milwaukee Instruments, Rocky Mount, NC, USA). All color and pH measurements were performed in triplicate for each sampled package, and the results were expressed as mean ± standard deviation to assess the antimicrobial activity and quality preservation effects of the CV and *t*CN surface coatings.

### 2.6. Statistical Analysis

All experiments were performed with a minimum of three independent replicates (*n* ≥ 3), with five replicates employed for mechanical, barrier, and minced pork TVC analyses. Data are presented as the mean ± standard deviation (SD). Statistical analysis was performed using SPSS software (Version 28.0, IBM Corp., Armonk, NY, USA). Normality of data distribution was assessed using the Shapiro–Wilk test, and homogeneity of variances was verified using Levene’s test. As several datasets deviated from normal distribution or exhibited heterogeneity of variances—which is common in complex material characterization and food preservation studies—non-parametric statistical methods were primarily employed to ensure robust and reliable comparisons.

For the comparison of means across multiple independent groups (e.g., the effect of packaging type on TVC at different storage times, desorption kinetic parameters at different temperatures, mechanical properties, oxygen barrier properties, antioxidant activity, antibacterial inhibition zones, and color parameters), the Kruskal–Wallis one-way analysis of variance (ANOVA) by ranks was employed. This non-parametric test is suitable for comparing three or more independent groups without assuming a normal distribution. When the Kruskal–Wallis test indicated statistically significant differences (*p* < 0.05), Dunn’s post hoc test with Bonferroni correction was applied for pairwise comparisons to identify which specific groups differed significantly. This approach was consistently applied to all datasets presented in Table 1, Table 2, Table 3, Table 4, Table 5 and Table 6.

For comparisons involving only two independent groups (e.g., specific pairwise comparisons of in vitro antimicrobial or antioxidant assays between coated and uncoated materials), the Mann–Whitney U test (the non-parametric equivalent of the independent samples *t*-test) was used.

The significance level for all statistical tests was set at *p* < 0.05. Different superscript letters (e.g., ^a^, ^b^, ^c^) or symbols (e.g., ^†^, ^‡^, ^§^) within the same row or column of data tables indicate statistically significant differences between groups based on the Kruskal–Wallis and Dunn’s post hoc analyses, as specified in each table caption. This rigorous non-parametric approach ensures that the conclusions drawn regarding the performance of the active packaging systems are robust and not biased by potential violations of parametric test assumptions.

## 3. Results

### 3.1. Physicochemical Characterization of Surface Coated PET-CV and PET-tCN

#### 3.1.1. XRD

**Figure 1:**
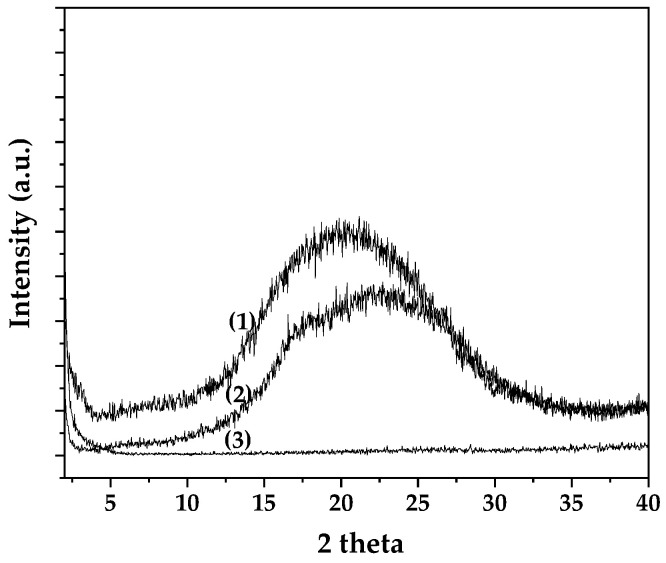
XRD plots of (1) pure PET, (2) PET-CV, and (3) PET-*t*CN.

The XRD patterns of pure PET, PET-CV, and PET-*t*CN are presented in Figure 1 for comparative analysis.

As shown in Figure 1, pure PET exhibited a broad diffraction peak centered at approximately 20° 2θ, characteristic of its predominantly amorphous structure [34,35]. Surface modification with CV (PET-CV) resulted in a slight attenuation of this amorphous peak, while the PET-*t*CN sample displayed a more notable reduction in the intensity of the amorphous halo. These observations suggest that the presence of the biocide coatings, particularly *t*CN, may influence the near-surface region of the PET, potentially through molecular interactions that affect polymer chain packing. However, it is important to note that conventional XRD probes the bulk of the material (several micrometers in depth), and the coatings themselves are only ~25–40 µm thick (Section 3.1.7). Therefore, the observed changes in the diffraction pattern cannot be definitively attributed to surface-specific phenomena such as “surface crystallization” without additional surface-sensitive analyses (e.g., grazing-incidence XRD) [36,37]. The results primarily confirm the successful deposition of the coatings and suggest some degree of interaction with the PET surface, which is consistent with the FTIR and surface energy data presented in Section 3.1.2 and Section 3.1.6.

#### 3.1.2. ATR-FTIR

**Figure 2:**
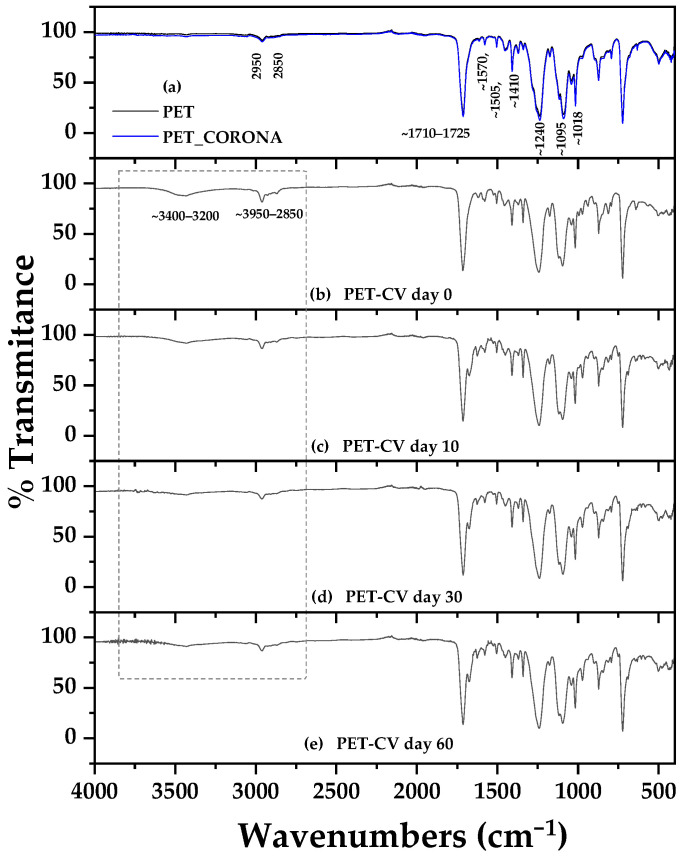
ATR-FTIR spectra of (**a**) pure PET (black line) and corona-treated PET (blue line), (**b**) PET-CV on day 0, (**c**) PET-CV on day 10, (**d**) PET-CV on day 30, and (**e**) PET-CV on day 60.

**Figure 3:**
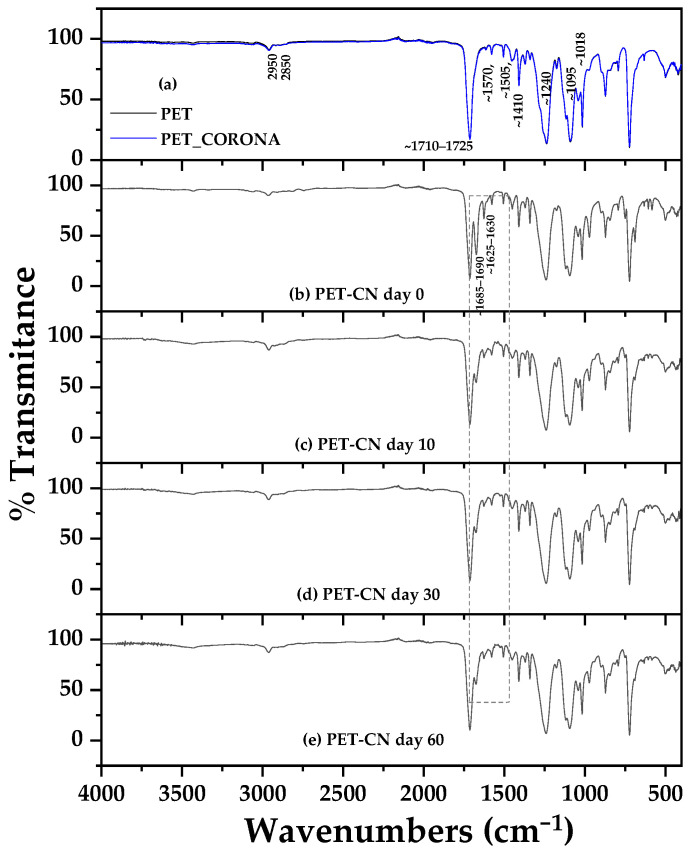
ATR-FTIR spectra of (**a**) pure PET (black line) and corona-treated PET (blue line), (**b**) PET-CN on day 0, (**c**) PET-CN on day 10, (**d**) PET-CN on day 30, and (**e**) PET-CN on day 60.

ATR-FTIR spectra of pure PET, corona-treated PET (PET-co), and PET-CV samples stored for 0, 10, 30, and 60 days are shown in Figure 2 for comparative analysis. Corresponding spectra for PET-*t*CN (PET-*t*CN) under the same storage conditions are presented in Figure 3.

The ATR-FTIR spectrum of unmodified PET (see Figure 2a and Figure 3a black line) exhibited characteristic vibrational bands of polyethylene terephthalate. The strong, sharp peak at approximately 1715 cm^−1^ corresponds to the C=O stretching vibration of the ester carbonyl group [34]. Aromatic ring vibrations were observed at 1570, 1505, and 1455 cm^−1^, attributed to C=C stretching of the phenyl rings [44]. The asymmetric C–O–C stretching vibration of the ester linkage appeared near 1240 cm^−1^, while the symmetric stretch was noted around 1095 cm^−1^. Fingerprint region bands at 870 cm^−1^ and 725 cm^−1^ confirmed the presence of 1,4-disubstituted benzene rings via aromatic C–H out-of-plane bending modes [35]. Aliphatic C–H stretching vibrations of methylene groups were weakly visible in the 2950–2850 cm^−1^ range.

Corona treatment (Figure 2a and Figure 3a blue line) did not significantly alter the primary PET bands, indicating that the bulk polymer structure remained intact. However, a subtle increase in broad absorption in the 3200–3500 cm^−1^ region suggests the introduction of surface hydroxyl (–OH) groups due to plasma oxidation, a known effect of corona discharge that enhances surface polarity and wettability [36,37,38].

Successful surface coating with CV was confirmed by the appearance of characteristic CV bands in the PET-CV spectra (Figure 2b–e). A prominent broad band in the 3400–3200 cm^−1^ region corresponds to the phenolic O–H stretching vibration of CV [22]. The increased intensity in the 2950–2850 cm^−1^ range is attributed to overlapping aliphatic C–H stretches from CV’s isopropyl and methyl groups. Additionally, the region between 1600 and 1500 cm^−1^ shows enhanced aromatic C=C stretching contributions from CV’s phenolic ring [39]. The persistence of these CV-specific bands throughout the 60-day storage period indicates stable surface retention of CV, with no significant desorption or degradation under ambient conditions.

Similarly, the spectra of PET-CN (Figure 3b–e) confirmed the successful coating of trans-cinnamaldehyde (*t*CN). Key diagnostic bands for *t*CN include the aldehyde C–H stretching doublet at approximately 2820 and 2730 cm^−1^ (Fermi resonance) and the conjugated C=O stretch at ~1685 cm^−1^, which is distinct from and appears as a shoulder to the PET ester carbonyl peak at 1715 cm^−1^ [44]. The C=C stretching vibration of the conjugated alkene is observed at ~1630 cm^−1^, and the characteristic trans-alkene out-of-plane bending appears as a strong band near 970 cm^−1^ [27]. These *t*CN-related bands remained clearly visible over the entire 60-day period, demonstrating that *t*CN was also effectively anchored to the corona-activated PET surface.

The absence of new covalent bond formation (e.g., ester or ether peaks) in both PET-CV and PET-*t*CN spectra suggests that both biocides are primarily physically adsorbed rather than chemically grafted. The observed stability of the coatings over 60 days under ambient storage conditions is encouraging and suggests that the interactions are sufficient for practical applications under similar conditions. These interactions likely involve a combination of hydrogen bonding, dipole–dipole interactions, and van der Waals forces between the polar functional groups of the biocides (phenolic --OH of CV, aldehyde C=O of *t*CN) and the oxygen-rich, hydrophilic surface created by corona treatment [27,38]. This interpretation is consistent with the FTIR data, although further studies (e.g., XPS, surface energy measurements) would be needed to definitively characterize the exact binding energies and interaction mechanisms. It should also be noted that while the 60-day stability under ambient conditions is promising, performance under varied temperature, humidity, or mechanical stress conditions may differ and requires further investigation.

#### 3.1.3. Desorption Kinetics

**Figure 4:**
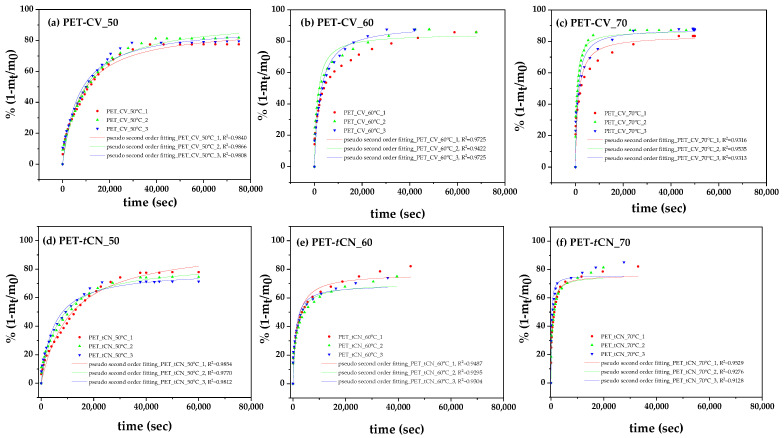
Desorption kinetic profiles of (**a**) CV from PET-CV and (**b**) *t*CN from PET-*t*CN at 50, 60, and 70 °C. Symbols represent experimental data; solid lines represent the pseudo-second-order kinetic model fit.

The desorption kinetics of CV and *t*CN from the surface-coated PET-CV and PET-*t*CN packages were studied at 50, 60, and 70 °C to evaluate the thermal stability and release behavior of the bioactive coatings. The normalized release fraction $q_{t}$ was plotted against time for each temperature, as shown in Figure 4.

The experimental data were fitted using the pseudo-second-order kinetic model (Equation (3)). As shown by the high $R^{2}$ values (Table 1), this model provided an excellent fit in all cases. The derived kinetic parameters—the rate constant $k_{2}$ and the equilibrium desorption fraction $q_{e}$—are summarized in Table 1.

**Figure 5:**
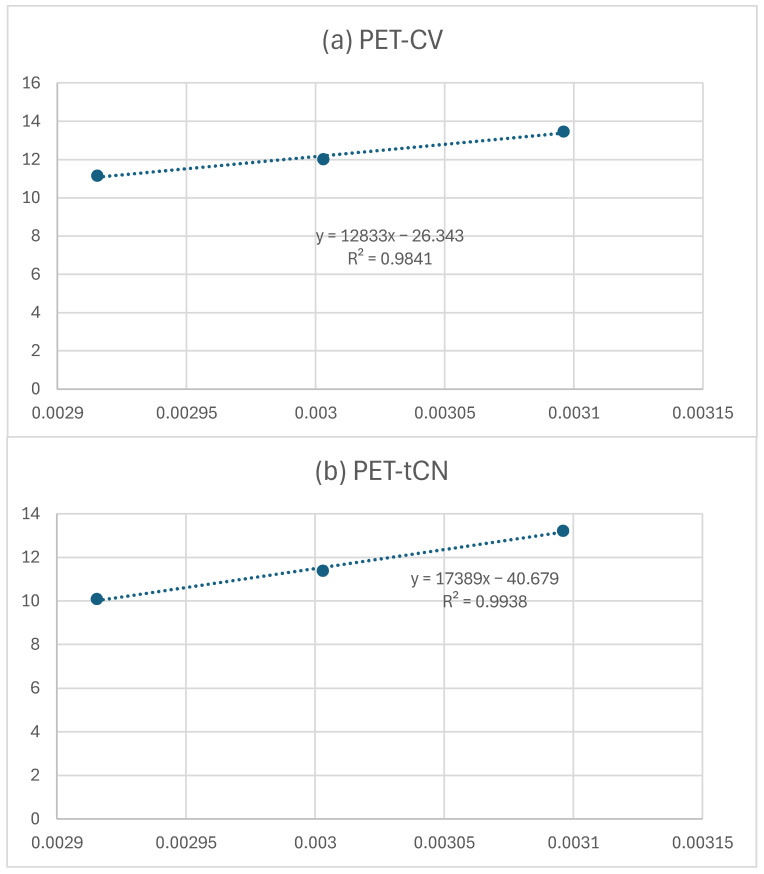
Arrhenius plots of ln $k_{2}$ versus 1/T for CV and *t*CN desorption from PET-CV and PET-*t*CN, respectively.

From the linear fits shown in Figure 5 and the corresponding linear equations, the calculated slopes were used in conjunction with Equation (5) to determine the desorption energies (E_0,des_) of CV and *t*CN. The estimated values were 94.9 kJ/mol (22.7 kcal/mol) for PET-CV and 128.0 kJ/mol (30.6 kcal/mol) for PET-*t*CN.

It is important to note that these desorption measurements were performed at elevated temperatures (50–70 °C) to accelerate the release process and enable kinetic analysis within a practical timeframe. While this provides valuable comparative information about the relative release behavior and interaction strengths of the two coatings, the absolute release rates and kinetics under actual food storage conditions (e.g., 4 °C for refrigerated meat or 23 °C for ambient olive storage) would be substantially slower. Therefore, these data should be interpreted as an indication of relative release tendencies rather than as a direct quantitative model of food-contact release.

The desorption kinetic study provides critical insights into the thermal stability and release behavior of CV and *t*CN from corona-treated PET surfaces. The higher k_2_ values observed for *t*CN across all temperatures indicate a faster release rate compared to CV, which is consistent with its higher activation energy for desorption (128.0 vs. 94.9 kJ/mol). This suggests that *t*CN is more strongly bound to the surface, requiring more energy for release, yet its kinetic profile shows a higher initial rate constant. Conversely, the slower release of CV, governed by different interfacial interactions, aligns with its lower E_0,des_ value and supports its potential for a more moderate preservation efficacy.

The kinetic findings are consistent with the structural insights from XRD and FTIR analyses. The XRD results showed a reduction in the amorphous halo for PET-*t*CN, which may reflect molecular interactions between *t*CN and the PET surface, potentially involving π-π stacking or dipole–dipole interactions with the polymer backbone [36,37]. This ordered interfacial layer may facilitate a more uniform but initially faster release of *t*CN under thermal activation. In contrast, CV’s interaction appears to be dominated by hydrogen bonding with the corona-induced hydroxyl groups, as evidenced by the persistent O–H stretching band in the FTIR spectra over 60 days [22,39]. This hydrogen-bonding network likely contributes to the different desorption energy and release kinetics observed for CV.

The high q_e_ values (72–91%) confirm that a substantial fraction of both biocides remains releasable under thermal conditions, suggesting potential for sustained release during storage and distribution, although confirmation under actual food-contact conditions would be needed. The slightly lower q_e_ for *t*CN may reflect its partial integration into the surface-modified PET structure, limiting complete desorption within the experimental timeframe—a phenomenon supported by the XRD observation of reduced amorphous scattering.

From a practical standpoint, the differing release profiles of CV and *t*CN offer flexibility in designing active packaging for specific food applications. Based on the relative release behaviors observed at elevated temperatures, *t*CN’s faster initial release may be suitable for highly perishable products requiring immediate antimicrobial protection, while CV’s different kinetic profile could benefit products with varying shelf-life requirements. However, it should be emphasized that these suggestions are based on comparative kinetic data, and validation under actual food storage conditions (e.g., 4 °C for refrigerated products, 23 °C for ambient storage) would be needed to confirm these proposed application-specific advantages. Moreover, the use of corona treatment as a scalable surface activation method ensures that these release characteristics can be reliably reproduced in industrial settings, enhancing the translational potential of this approach. These distinct kinetic profiles suggest that PET-CV, with its slower release, is better suited for long-term preservation applications, while PET-*t*CN, with its faster initial release, is ideal for short-term, high-risk perishable foods, pending confirmation under real-world conditions.

In summary, the desorption kinetics, combined with complementary physicochemical characterizations, indicate that corona treatment effectively anchors CV and *t*CN onto PET surfaces through distinct interaction mechanisms. These interactions appear to dictate the release behavior and thermal stability of the biocides under the tested conditions, providing a basis for the rational design of active PET packaging with tailored preservation performance for diverse food systems, though further validation under real-world conditions is recommended.

#### 3.1.4. SEM Images

**Figure 6:**
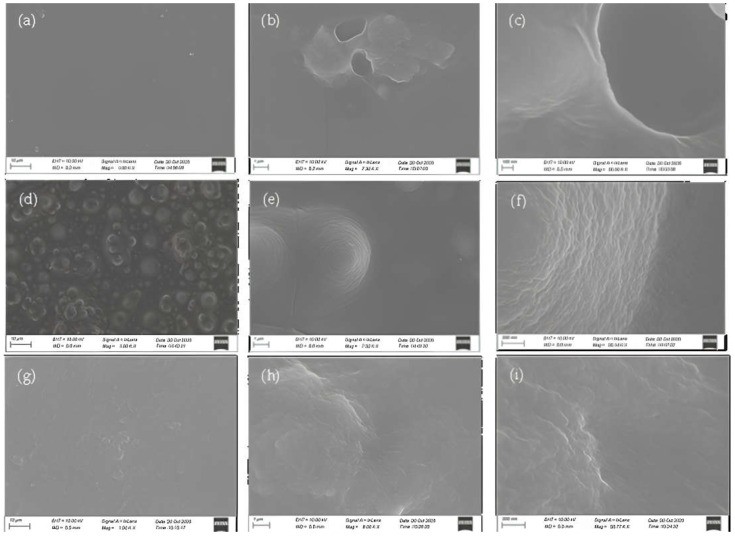
SEM micrographs of (**a**–**c**) uncoated PET (Ref), (**d**–**f**) PET-CV, and (**g**–**i**) PET-*t*CN at low (1.00 kX) (**a**,**d**,**g**), medium (7.30 kX) (**b**,**e**,**h**), and high (55.00 kX) (**c**,**f**,**i**) magnifications.

Scanning electron microscopy (SEM) was employed to investigate the surface morphology of the uncoated PET (Ref), CV-coated (PET-CV), and *t*CN-coated (PET-*t*CN) films. The SEM images were captured across a range of magnifications (100× to 55.00 kX) and are presented in Figure 6.

The uncoated PET (Ref) exhibited a surface texture with some fine structural features visible at higher magnifications (Figure 6a–c). It should be noted that such features on commercial polymer surfaces can be influenced by sample preparation (e.g., gold coating), imaging conditions, or charging artifacts, and caution is needed in interpreting the absolute native morphology. Therefore, detailed nanoscale morphological interpretations should be considered with appropriate caution.

In contrast, the PET-CV sample (Figure 6d–f) displayed a visibly denser and more uniform surface layer compared to the uncoated reference, consistent with the presence of a continuous coating. The surface appears smoother and less textured than the Ref sample, suggesting that the CV coating effectively covers the underlying PET surface.

The PET-*t*CN sample (Figure 6g–i) showed an intermediate appearance, with a surface layer that appears less continuous than PET-CV but still distinctly different from the uncoated control. Some minor surface irregularities are visible, which may reflect differences in the coating formation or drying behavior of *t*CN compared to CV.

These images confirm the successful deposition of both coatings and suggest differences in their surface coverage and uniformity. The more continuous appearance of the PET-CV coating correlates with its slower, more sustained release kinetics observed in the desorption studies (Section 3.1.3), as a denser coating may create a more tortuous diffusion path. Conversely, the slightly less uniform appearance of the PET-*t*CN coating is consistent with its faster initial release rate, as a less continuous layer might facilitate more rapid mass transfer. However, these morphology-function correlations should be considered as qualitative observations rather than proven mechanisms, as surface imaging alone cannot definitively establish release pathways.

#### 3.1.5. AFM Surface Topography Analysis

**Figure 7:**
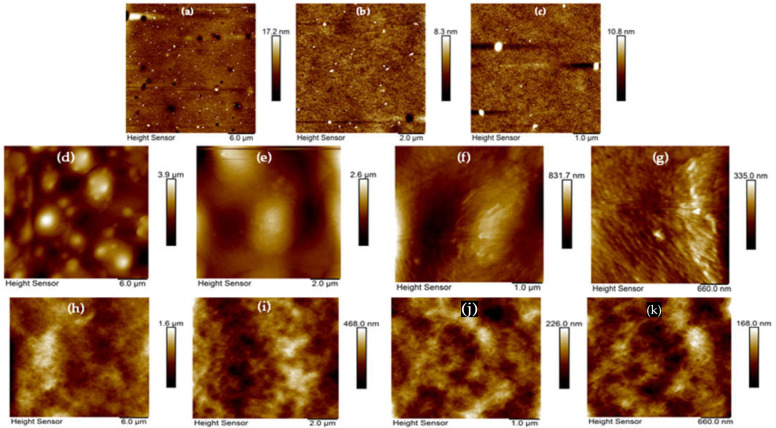
AFM topographical maps (2D and 3D) of (**a**–**c**) uncoated PET (Ref), (**d**–**g**) PET-CV, and (**h**–**k**) PET-*t*CN, scanned over an area of 1 µm × 1 µm. For each sample: left column shows 2D height map, middle column shows 3D rendered surface, and right column shows corresponding cross-sectional height profile. Average roughness (Ra) values are provided in the text.

Atomic force microscopy (AFM) was performed to assess the surface topography of the uncoated PET (Ref), CV-coated (PET-CV), and *t*CN-coated (PET-*t*CN) samples. All images were acquired over a scan area of 1 µm × 1 µm, and the average roughness (Ra) was calculated for each sample from the topographical maps. Representative AFM images and corresponding cross-sectional height profiles are presented in Figure 7.

Uncoated PET exhibited an exceptionally smooth and uniform surface, with an average roughness (Ra) of approximately 1 nm. This confirms the absence of significant topographical features on the native PET surface that could influence coating adhesion or release behavior.

The PET-CV sample presented a highly irregular topography with considerable variation in surface features. The Ra values ranged from approximately 30–80 nm across most of the scanned area, with some larger aggregated structures reaching local heights corresponding to Ra values up to 400 nm (Figure 7d). This significant variability suggests the formation of aggregates or non-uniform coating deposition, which may affect the consistency of the coating’s barrier and release properties.

The PET-*t*CN sample displayed a more uniformly rough surface, with Ra values ranging from approximately 18 nm to 154 nm across the scanned areas. This indicates the presence of densely distributed nanoscale features and suggests a more homogeneous coating layer compared to PET-CV.

These AFM observations are consistent with the SEM findings (Section 3.1.4) and provide quantitative confirmation of the distinct surface topographies of the two coatings. The smoother, more uniform surface of uncoated PET confirms that the observed roughness on the coated samples originates from the deposited biocide layers rather than the substrate itself.

Qualitatively, the different topographies may relate to the distinct release behaviors observed in the desorption studies (Section 3.1.3). The more irregular surface of PET-CV could create a more tortuous diffusion path, potentially contributing to its slower release kinetics. Conversely, the uniformly rough surface of PET-*t*CN might facilitate more rapid initial mass transfer. However, these morphology-function correlations should be considered as qualitative observations rather than statistically validated mechanisms. A definitive correlation between specific topographical parameters and release kinetics would require more extensive analysis across multiple samples and scan areas, coupled with rigorous statistical modeling.

The AFM results also complement the surface energy measurements (Section 3.1.6), as the different topographies may influence the effective surface area available for interfacial interactions. The uniformly rough surface of PET-*t*CN, combined with its higher surface energy (50 ± 2 dynes/cm), may promote better wetting and contact with food matrices, potentially contributing to its superior antimicrobial performance in the food preservation tests (Section 3.3 and Section 3.4).

In summary, AFM analysis quantitatively confirms the distinct surface topographies of CV- and *t*CN-coated PET. The smoother, uniform surface of PET-CV and the uniformly rough surface of PET-*t*CN provide valuable insights into the physical characteristics of the coatings. While these topographical differences are consistent with the observed functional performance, further studies would be needed to establish direct mechanistic correlations.

#### 3.1.6. Surface Energy Analysis

The surface energy of the different PET films, as determined by ACCU DYNE TEST™ Marker Pens, is presented in Table 2.

The unmodified PET surface exhibited a surface energy of 40 ± 2 dynes/cm, consistent with its relatively inert and hydrophobic nature. Corona treatment (PET-co) resulted in a dramatic and statistically significant (*p* < 0.05) increase in surface energy to 58 ± 2 dynes/cm. This substantial increase of 18 dynes/cm directly confirms the successful surface activation via the introduction of polar functional groups, which enhances wettability and provides binding sites for the subsequent biocide coatings. After coating, the surface energy of PET-CV and PET-*t*CN was measured at 46 ± 2 dynes/cm and 50 ± 2 dynes/cm, respectively. These values, which are significantly higher than the unmodified PET but lower than the freshly corona-treated surface, indicate that the biocides have successfully formed a coating, presenting their own characteristic surface chemistry. The higher surface energy of PET-*t*CN compared to PET-CV suggests a more polar surface, which may correlate with its different molecular structure and interaction with the activated layer.

#### 3.1.7. Coating Thickness

The thickness of the CV and *t*CN coatings was estimated using a gravimetric method on circular substrates. Five circular PET coupons with a diameter of 3.8 cm (radius = 1.9 cm) were weighed individually. They were then corona-treated and coated with the biocide following the procedure in Section 2.2. After the 24 h stabilization period, the coated coupons were weighed again. The mass of the applied coating was calculated by subtracting the initial mass from the final mass. The coating thickness (h) was estimated using the formula derived from the geometric relationship: mass = density × volume, and for a thin coating on a flat substrate, volume = area × height. Thus, h = m / (ρ × A), where m is the average mass of the coating (g), ρ is the density of the biocide (CV: 0.977 g/cm^3^; *t*CN: 1.05 g/cm^3^), and A is the coated surface area (A = πr^2^ = π × (1.9 cm)^2^ ≈ 11.34 cm^2^). The calculated parameters and final thickness are presented in Table 3. The thickness is reported in micrometers (µm) as mean ± standard deviation.

The gravimetric analysis revealed that both coatings formed a layer on the micrometer scale. The average thickness of the CV coating was 25.8 ± 3.2 µm, while the *t*CN coating was significantly thicker, at 39.0 ± 4.5 µm. This difference may be attributed to the different masses applied and the densities and spreading behaviors of the two liquids on the activated PET surface. The formation of such a microscale layer is consistent with the preservation of transparency (Section 3.1.8) and the minimal impact on the bulk mechanical properties (Section 3.2.1).

#### 3.1.8. Optical Transparency

**Figure 8:**
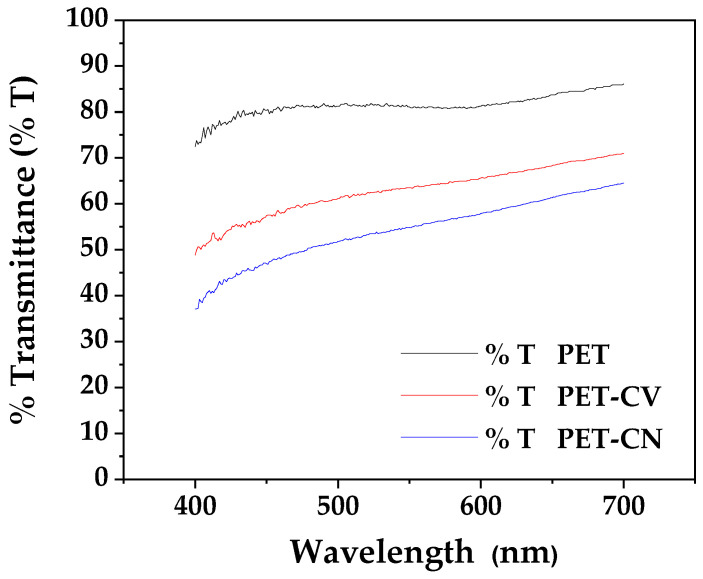
UV-Vis transmittance spectra of unmodified PET, PET-CV, and PET-*t*CN films over the visible wavelength range (400–700 nm).

The UV-Vis transmittance spectra of unmodified and surface-coated PET films over the 400–700 nm range are presented in Figure 8.

The unmodified PET film showed high transparency, with an average transmittance of approximately 90% across the visible range (400–700 nm). After surface coating, both modified films exhibited a reduction in optical transmittance. The PET-CV film showed an average transmittance of approximately 82%, while the PET-*t*CN film maintained a higher average transmittance of approximately 87%. The slight decrease in transparency, particularly for PET-CV, can be attributed to light scattering caused by the surface coating morphology. As observed in the AFM analysis (Section 3.1.5), PET-CV exhibited a rougher and more irregular topography, which may contribute to increased light scattering. Despite this minor reduction on the coated surfaces, it is important to note that the sides of the PET packages remain uncoated and fully transparent, ensuring that overall product visibility is not compromised for the consumer.

### 3.2. Packaging Properties of Modified PET-CV and PET-tCN

#### 3.2.1. Tensile Properties

The tensile properties of the unmodified and surface-modified PET films are summarized in Table 4. A Kruskal–Wallis non-parametric test was employed to assess statistical significance, followed by Dunn’s post hoc test for pairwise comparisons, with significance set at *p* < 0.05.

The statistical analysis reveals a significant effect of the surface modification on the elastic modulus (*p* < 0.001). The unmodified PET film exhibited the highest stiffness (1448.98 ± 58.04 MPa^a^). Both biocide-coated films, PET-CV and PET-*t*CN, showed a statistically similar and significant reduction in stiffness (1247.34 ± 44.52 MPa^b^ and 1156.42 ± 96.75 MPa^b^, respectively), representing a decrease of approximately 14–20%. This reduction is likely attributable to the surface coatings, which may introduce a more compliant interfacial layer or slightly disrupt the polymer surface structure, thereby affecting the initial elastic response under tensile load.

In contrast, the ultimate tensile strength and elongation at break—key indicators of the material’s load-bearing capacity and ductility—were not significantly altered by the surface treatments (*p* = 0.558 and *p* = 0.192, respectively). All three film types belonged to the same statistical group (^a^) for these properties. This decoupling of mechanical behavior is crucial for packaging applications. It indicates that while the corona treatment and biocide coating process modulates the material’s rigidity, it preserves the core mechanical integrity and toughness of the PET substrate. The coatings, therefore, impart the desired active functionality without critically compromising the essential mechanical performance required for handling and containment.

#### 3.2.2. Oxygen Barrier Properties

The oxygen barrier performance of the surface-coated PET films was quantitatively assessed, and the key parameters are summarized in Table 5. The results demonstrate that the application of CV and *t*CN coatings via corona activation significantly enhances the oxygen barrier of standard PET packaging.

As shown in Table 5, the oxygen transmission rate (OTR) of pure PET was measured at 323.7 ± 73.3 cm^3^/(m^2^·day), characteristic of its moderate oxygen barrier properties suitable for many food packaging applications [34]. Surface modification with CV (PET-CV) resulted in a statistically significant (*p* < 0.05) reduction in OTR to 125.0 ± 20.0 cm^3^/(m^2^·day), representing a 61% improvement in barrier performance. More remarkably, the PET-*t*CN coating achieved an OTR of 64.2 ± 8.0 cm^3^/(m^2^·day), corresponding to an 80% reduction compared to pure PET, indicating substantially enhanced barrier properties.

This improvement in oxygen barrier was further confirmed by calculating the oxygen permeability coefficient (PeO_2_), which normalizes the transmission rate to film thickness. The PeO_2_ values followed the same statistical trend, decreasing from 7.49 × 10^−9^ cm^2^/s for pure PET to 2.89 × 10^−9^ cm^2^/s for PET-CV and 1.49 × 10^−9^ cm^2^/s for PET-*t*CN. The superior barrier performance of PET-*t*CN is particularly noteworthy and can be attributed to several factors related to the coating’s physical and chemical properties.

The enhanced barrier effect is likely due to a combination of mechanisms. First, the surface coatings create an additional diffusive path for oxygen molecules, effectively increasing the tortuosity of the permeation pathway. Second, as suggested by the XRD results (Section 3.1.1), the *t*CN coating may induce a more ordered interfacial region on the PET surface, potentially creating a denser packing that hinders gas diffusion [36,37]. The stronger interaction of *t*CN with the corona-activated PET surface, evidenced by the desorption kinetics showing higher activation energy for *t*CN release, suggests a more stable and possibly more uniform coating layer that provides a consistent barrier.

Furthermore, the aromatic structure of *t*CN, with its extended π-conjugation system, may create a more effective molecular barrier to oxygen permeation compared to the less planar CV molecule. This structural advantage, combined with the controlled release kinetics that maintain coating integrity over time, ensures sustained barrier performance throughout the product’s shelf life.

The improved oxygen barrier properties of the coated PET films have significant implications for food preservation. By reducing oxygen permeation, these active packages can effectively delay oxidative rancidity in lipid-containing foods like pork mince and prevent oxidative degradation of pigments and nutrients in products like table olives. This barrier enhancement, combined with the antioxidant and antimicrobial activities demonstrated in subsequent sections, creates a multi-functional packaging system that addresses multiple food spoilage mechanisms simultaneously.

The fact that these barrier improvements were achieved through a simple surface coating process without altering the bulk PET properties represents a significant advancement in sustainable active packaging technology. (Recyclability, however, remains to be verified.). This approach maintains the mechanical integrity and transparency of PET while adding functional properties that extend food shelf life, aligning with circular economy principles by enhancing the value and performance of existing packaging materials.

#### 3.2.3. Antioxidant Activity

The in vitro antioxidant activity, quantified by the EC_50_ value from the DPPH assay, revealed a clear and statistically significant (*p* < 0.05) bioactivity gradient: PET-CV (13.5 ± 1.1 mg/mL) > PET-*t*CN (52.9 ± 4.7 mg/mL) >> Pure PET (0.0 mg/mL) (Table 5). The complete lack of activity for pure PET confirms that the antioxidant capacity originates solely from the surface-anchored bioactive compounds.

The superior potency of CV is directly linked to its molecular structure and release kinetics. CV (C_10_H_14_O), a monoterpenoid phenol, acts primarily through its phenolic –OH group, which is an effective hydrogen donor for neutralizing free radicals [22]. In contrast, trans-cinnamaldehyde (*t*CN) possesses a conjugated system comprising an aromatic ring, a carbon–carbon double bond, and an aldehyde group (C_6_H_5_–CH=CH–CHO). While this structure also exhibits antioxidant activity, the lower EC_50_ of PET-CV indicates that CV’s phenolic hydroxyl group is more efficient at scavenging DPPH radicals under the tested conditions, a finding consistent with comparative studies on essential oil components [16,27].

Crucially, this enhanced activity is sustained by the release profile. The desorption kinetics (Section 3.1.3) demonstrated a controlled, temperature-dependent release for both compounds. The calculated activation energy for desorption (E^0^_des_) was lower for PET-CV than for PET-*t*CN, indicating that CV is released more readily under ambient conditions, thereby maintaining a higher effective concentration at the packaging–food interface. This controlled, sustained release from the surface ensures prolonged availability of active molecules, which is critical for long-term antioxidant protection against lipid oxidation in fatty foods like pork mince [31].

#### 3.2.4. Antibacterial Activity

**Figure 9:**
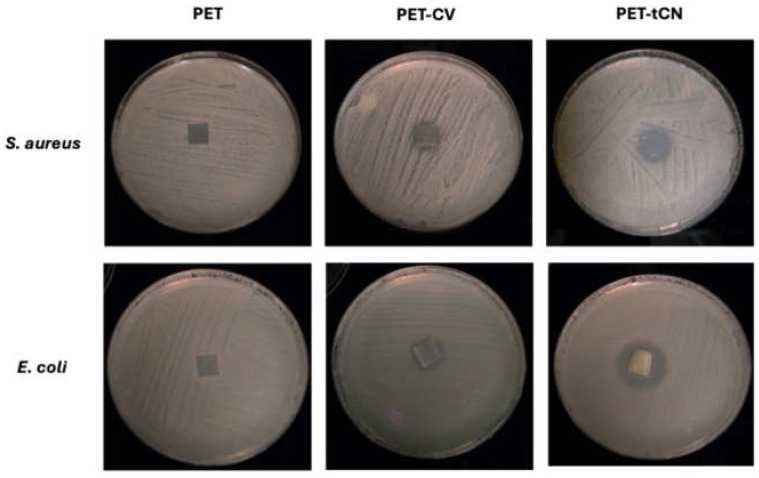
Representative images of the antibacterial activity of pure PET, PET-CV, and PET-tCN films against *S. aureus* and *E. coli* after 24 h incubation.

The in vitro antibacterial efficacy of the surface-modified PET films was assessed against *Staphylococcus aureus* (Gram-positive) and *Escherichia coli* (Gram-negative) by measuring the zone of inhibition surrounding the film (Table 5). As expected, the PET control exhibited no inhibitory activity against either bacterial strain (0.0 ± 0.0 mm), confirming that the polymer matrix is biologically inert and does not contribute to bacterial suppression. For surface modification, both PET-CV and PET-*t*CN films demonstrated significant antibacterial activity. Representative images of the inhibition zones are displayed in Figure 9. However, a marked difference in efficacy was observed between the two PET films. PET-CV films produced low inhibition zones of 4.0 ± 1.2 mm against *S. aureus* and 7.0 ± 0.5 mm against *E. coli*. In contrast, the PET-*t*CN films exhibited superior antimicrobial performance, achieving significantly larger inhibition zones of 8.0 ± 1.0 mm and 15.0 ± 1.0 mm, respectively. It should be noted that the disk diffusion assay measures the diffusible antimicrobial activity of the coatings and serves as a useful comparative screening tool. However, for surface-bound active packaging, this method may not fully represent contact-killing efficiency under real-world conditions. The subsequent food preservation tests (Section 3.3 and Section 3.4) provide a more realistic assessment of the packaging’s antimicrobial performance in contact with food matrices.

This high antimicrobial activity of PET-*t*CN can be attributed to the specific molecular mechanism of *trans*-cinnamaldehyde. In contrast to carvacrol, which acts mainly by disrupting the integrity of the bacterial cell membrane through its hydrophobic phenolic structure, *t*CN possesses a highly reactive acrolein group [44,45]. This electrophilic group can covalently bind to nucleophilic groups on essential bacterial proteins and nucleic acids via Schiff base formation, thereby inhibiting vital enzymatic systems and cell division processes, such as the function of the FtsZ protein [46,47]. This mechanism is known to be particularly effective against Gram-negative bacteria such as *E. coli*, as the small, lipophilic nature of the aldehyde allows it to penetrate the outer membrane porins and gain access to the cytoplasm [47].

### 3.3. Packaging Preservation Test-Preservation of Fresh Minced Pork

To assess the practical antimicrobial performance of the developed active packaging under real-world refrigeration conditions, a preservation test was conducted using fresh minced pork, a highly perishable food matrix prone to rapid microbial spoilage. The following table presents the evolution of Total Viable Count (TVC) over a 6-day storage period at 4 °C for meat packaged in unmodified PET, PET-CV, and PET-*t*CN containers.

The superior preservation efficacy of PET-*t*CN packaging, as evidenced by the statistically significant 2-log reduction in TVC compared to control PET after 6 days of refrigerated storage (Table 6), emerges as the functional culmination of the material’s engineered physicochemical properties. This enhanced performance can be directly attributed to the synergistic interplay between the corona-induced surface activation, the tailored release kinetics of *t*CN, and its inherent bioactivity. The corona treatment, confirmed by ATR-FTIR through the introduction of surface hydroxyl groups, provided the essential polar sites for the stable physical adsorption of *t*CN molecules [36,37]. Crucially, the desorption kinetic study (Section 3.1.3) revealed that *t*CN exhibited a higher activation energy for release (128.0 kJ/mol) compared to CV (94.9 kJ/mol), indicating stronger interfacial bonding to the activated PET surface. This stronger bonding does not preclude release but rather facilitates a more controlled and sustained delivery of the bioactive compound to the pork surface, as modeled by the pseudo-second-order kinetics. This sustained release profile is critical for long-term antimicrobial action, preventing the rapid depletion observed with more volatile compounds and maintaining an effective concentration at the food-packaging interface throughout storage [28,29]. The antimicrobial efficacy is further amplified by the intrinsic potency of *t*CN, which demonstrated superior in vitro activity against both *L. monocytogenes* and *E. coli* (Section 3.2.4), and its contribution to enhanced oxygen barrier properties (80% reduction in OTR, Section 3.2.2). The reduced oxygen permeation hinders the growth of aerobic spoilage microorganisms, creating a multi-hurdle preservation system. Furthermore, the uniform nano-roughness of the PET-*t*CN coating observed via AFM (Section 3.1.5) likely promotes greater interfacial contact with meat exudates, optimizing the transfer of *t*CN to the food matrix. In contrast, PET-CV showed intermediate performance, correlating with its different desorption energy, faster initial release kinetics, and slightly lower in vitro antimicrobial potency. The preservation results thus demonstrate the potential of repurposing corona treatment to anchor natural biocides, transforming inert PET into a multi-functional active packaging system where the release kinetics, dictated by surface interactions, contribute to extended shelf-life for perishable foods like fresh minced pork under the tested refrigerated conditions.

To comprehensively evaluate the impact of the active packaging systems on the physicochemical quality of fresh minced pork during refrigerated storage, surface color parameters (L, a, b*) and pH were monitored over the 6-day storage period at 4 °C. Color is the primary sensory attribute influencing consumer purchase decisions for fresh meat, with a* (redness) being particularly critical as it correlates with the oxidation state of myoglobin. The pH provides an indirect indicator of microbial metabolic activity and the onset of spoilage. The following table presents the evolution of these quality attributes for minced pork stored in unmodified PET (Control), PET-CV, and PET-*t*CN packages.

Values represent mean ± standard deviation of five independent replicates (*n* = 5). Different superscript letters within the same row (across time points for the same sample) indicate a statistically significant change over time (*p* < 0.05, Kruskal–Wallis test with Dunn’s post hoc test). Different superscript symbols (†, ‡, §) within the same column for the same parameter indicate significant differences between packaging types at that time point (*p* < 0.05).

The physicochemical quality parameters presented in Table 7 provide compelling evidence that the active packaging systems, particularly PET-*t*CN, effectively preserve the critical quality attributes of fresh minced pork during refrigerated storage, aligning perfectly with the microbiological findings in Table 6. The most striking and commercially relevant finding is the preservation of the red color (a* value), which is the primary determinant of consumer acceptance for fresh meat due to the susceptibility of oxymyoglobin to oxidation into brown metmyoglobin [34,35]. The unmodified PET (Control) samples exhibited a dramatic and statistically significant (*p* < 0.05) decline in a* values, plummeting from 14.80 at Day 0 to just 5.10 by Day 6, representing a 65.5% loss in redness indicative of severe oxidative spoilage. This deterioration was accompanied by significant darkening (decrease in L), a rise in yellowness (b) from lipid oxidation byproducts, and a sharp pH increase from 5.60 to 6.65, the latter being a classic indicator of microbial protein degradation and the proliferation of psychrotrophic spoilage bacteria [43]. In contrast, the PET-CV packaging provided significant, though intermediate, protection, with the a* value decreasing to 9.20 by Day 6 (a 37.8% loss), and the pH rising only to 6.15. This substantial improvement over the control correlates directly with the 61% reduction in oxygen transmission rate (OTR) for PET-CV (Section 3.2.2) and its controlled release of the antioxidant and antimicrobial CV, which together slow myoglobin oxidation and suppress microbial metabolism [22]. Most remarkably, the PET-*t*CN packaging demonstrated outstanding holistic preservation. The a* value decreased only modestly to 12.80 by Day 6 (just a 13.5% loss), L* remained stable, b* showed no significant change, and pH increased by a mere 0.10 units to 5.70, remaining within the range of fresh meat. This exceptional performance provides direct evidence of the synergistic effects of the *t*CN coating: its superior 80% reduction in OTR (Section 3.2.2) creates a low-oxygen environment that stabilizes oxymyoglobin, while its potent, sustained antimicrobial activity—driven by a high desorption energy (128.0 kJ/mol) and a multi-target mechanism involving the reactive aldehyde group [46,47]—almost completely suppresses microbial metabolism, preventing alkalinization. The uniform nano-roughness of the PET-*t*CN coating (AFM, Section 3.1.5) likely further enhances these effects by ensuring uniform surface coverage and efficient compound transfer. Thus, by combining superior oxygen barrier properties, potent antimicrobial activity, controlled release kinetics, and favorable surface morphology, the PET-*t*CN packaging system delivers holistic preservation of fresh minced pork, maintaining both microbiological safety (Table 6) and critical sensory attributes throughout refrigerated storage, and conclusively validating the core innovation of this scalable technology.

### 3.4. Packaging Preservation Test-Preservation of Table Olives

To evaluate the antimicrobial efficacy of the surface-modified PET packaging under ambient storage conditions, a preservation test was conducted using ready-to-eat table olives over a 21-day period. The total viable count (TVC) was monitored at weekly intervals to assess the ability of the CV and *t*CN coatings to inhibit microbial growth and extend the shelf-life of a non-perishable, brine-free food product. The following table presents the mean TVC values (log_10_ CFU/olive) for olives stored in unmodified PET, PET-CV, and PET-*t*CN packages at 23 ± 2 °C.

The results from the olive preservation test, presented in Table 8, strongly reinforce the superior performance of the *t*CN-based active packaging observed in the minced meat study, demonstrating its efficacy in a different food matrix and under ambient storage conditions. All packages started with a uniform and low initial microbial load (*p* = 1.000), confirming that any subsequent differences in TVC are directly attributable to the packaging material’s activity.

From Day 7 onwards, a clear and statistically significant (*p* < 0.001) divergence in microbial growth kinetics was observed between the three packaging systems. The control PET packages allowed rapid microbial proliferation, reaching a high TVC of 8.32 log_10_ CFU/olive by Day 21, indicative of substantial spoilage. This uninhibited growth is expected, as unmodified PET provides no active barrier against microbial contamination.

In contrast, the PET-CV packaging demonstrated a significant inhibitory effect, maintaining a TVC approximately 0.5–1.0 log_10_ CFU/olive lower than the control throughout the storage period. This result confirms that the CV coating imparts functional antimicrobial activity to the PET surface. The reduction is likely due to the controlled release of CV, as characterized by its desorption kinetics (Section 3.1.3), and its membrane-disrupting mode of action [22,23], which can effectively suppress a broad spectrum of spoilage microorganisms. The intermediate performance of PET-CV aligns perfectly with its moderate in vitro antibacterial activity (Section 3.2.4) and its physicochemical properties, including its dense but irregular surface morphology observed in SEM and AFM (Section 3.1.4 and Section 3.1.5).

Most notably, the PET-*t*CN packaging exhibited the most potent and sustained antimicrobial effect. By Day 21, the TVC of olives stored in PET-*t*CN was 5.97 log_10_ CFU/olive, which represents a highly significant reduction of approximately 2.35 log cycles compared to the unmodified PET control (8.32 log_10_ CFU/olive) and 1.79 log cycles compared to PET-CV (7.76 log_10_ CFU/olive). This superior performance mirrors the findings from the minced pork test and can be attributed to the same synergistic factors identified throughout this study:

Strong Surface Anchoring: The stable adsorption of *t*CN to the corona-activated PET surface, confirmed by its high desorption energy (128.0 kJ/mol) and persistent FTIR signals, ensures the coating’s longevity.

Controlled Release Kinetics: The pseudo-second-order kinetics provide a sustained delivery of the bioactive compound to the olive surface, preventing rapid depletion [28,29].

Intrinsic Bioactivity: The multi-target antimicrobial mechanism of *t*CN, involving its highly reactive aldehyde group, is particularly effective [46,47], as reflected in its superior in vitro inhibition zones (Section 3.2.4).

Enhanced Oxygen Barrier: The 80% reduction in OTR for PET-*t*CN (Section 3.2.2) creates a modified atmosphere within the package, limiting the growth of aerobic spoilage organisms.

Favorable Surface Morphology: The uniform nano-roughness of the PET-*t*CN coating, observed via AFM, likely promotes greater interfacial contact with the olive surface, optimizing the transfer of *t*CN.

The results from this ambient-temperature study on table olives are particularly significant. They demonstrate that the developed active packaging is not only effective for high-risk refrigerated products like meat but also for shelf-stable products stored at room temperature. This broadens the potential application scope of this scalable technology. The ability of the PET-*t*CN system to dramatically slow microbial growth on a solid food matrix under these conditions underscores its potential as a powerful tool for reducing food waste and enhancing the safety and shelf-life of a wide variety of food products.

To comprehensively evaluate the impact of the active packaging systems on the physicochemical quality of table olives during ambient storage, surface color parameters (L, a, b*) and pH were monitored over the 21-day period. Color stability is a critical sensory attribute for consumer acceptance of table olives, with L* representing lightness/darkness, a* indicating greenness (negative values) to redness (positive values), and b* representing blueness (negative values) to yellowness (positive values). The pH provides an indirect indicator of microbial metabolic activity and potential acid migration from the active coatings. The following table presents the evolution of these quality attributes for olives stored in unmodified PET (Control), PET-CV, and PET-*t*CN packages at 23 ± 2 °C.

Values represent mean ± standard deviation of three independent replicates (*n* = 5). Different superscript letters within the same row (across time points for the same sample) indicate a statistically significant change over time (*p* < 0.05, Kruskal–Wallis test with Dunn’s post hoc test). Different superscript symbols (†, ‡, §) within the same column (comparing different samples at the same time point) indicate significant differences between packaging types (*p* < 0.05).

The physicochemical quality parameters presented in Table 9 provide compelling evidence that the active packaging systems, particularly PET-*t*CN, not only inhibit microbial growth but also preserve the intrinsic quality attributes of table olives during ambient storage. These results complement and mechanistically explain the microbiological findings discussed in Table 7.

The unmodified PET (Control) samples exhibited progressive and statistically significant (*p* < 0.05) color deterioration over the 21-day storage period. The decrease in lightness (L* from 30.235 to 25.703) and the substantial reduction in a* values (from 13.837 to 9.330) indicate significant browning and loss of the characteristic green hue of the olives. This discoloration is typical of oxidative degradation, where oxygen permeating through the conventional PET package promotes the oxidation of chlorophyll pigments and enzymatic browning by polyphenol oxidase (PPO) [34,35]. The increase in b* values (from 8.526 to 10.070) further confirms the development of yellow-brown discoloration.

In contrast, the PET-CV packaging provided moderate protection against color degradation. While some changes were observed, particularly in a* values which decreased to 10.918 by Week 3, the L* values remained relatively stable (no significant change from Day 0 to Week 3). This intermediate performance correlates well with the 61% reduction in oxygen transmission rate (OTR) for PET-CV reported in Section 3.2.2. The reduced oxygen permeation slows but does not completely halt the oxidative reactions responsible for color change.

Most remarkably, the PET-*t*CN packaging demonstrated exceptional color preservation. None of the color parameters (L, a, b*) showed statistically significant changes throughout the entire 21-day storage period. The a* values, indicative of the desirable green color, remained remarkably stable (13.837 at Day 0 vs. 14.040 at Week 3, *p* > 0.05). This outstanding performance provides direct visual evidence of the superior oxygen barrier properties of the *t*CN coating, which achieved an 80% reduction in OTR (Section 3.2.2). By dramatically limiting oxygen ingress, the PET-*t*CN package creates a modified atmosphere that effectively inhibits both pigment oxidation and enzymatic browning reactions. Furthermore, the uniform nano-roughness of the PET-*t*CN coating observed via AFM (Section 3.1.5) may contribute to a more complete and uniform surface coverage, enhancing its barrier efficacy.

The pH measurements provide an indirect but powerful indicator of microbial metabolic activity and chemical changes within the food matrix. The control PET samples showed a significant and progressive increase in pH from 4.207 at Day 0 to 4.620 at Week 3. This alkalinization is characteristic of microbial spoilage, where proteolytic bacteria break down proteins, producing ammonia and other alkaline compounds [43]. This pH trend aligns perfectly with the rapid microbial growth observed in the control samples in Table 5 (TVC reaching 8.32 log CFU/olive by Week 3).

The PET-CV packaging effectively stabilized the pH, with values remaining consistently around 4.58 from Week 1 through Week 3. This stability indicates successful suppression of microbial metabolism, consistent with the intermediate antimicrobial activity of CV observed in both the in vitro assays (Section 3.2.4) and the TVC results (Table 6). The controlled release of CV from the coating, governed by its pseudo-second-order kinetics (Section 3.1.3), maintains an effective concentration of the antimicrobial compound at the olive surface, inhibiting bacterial proliferation and the associated metabolic byproducts.

The PET-*t*CN samples exhibited a striking and statistically significant decrease in pH, from 4.207 at Day 0 to 3.450 at Week 3. This acidification trend is distinctly different from the control and PET-CV samples and can be attributed to two synergistic mechanisms:

Release of Acidic Compounds: *t*CN itself, while not a strong acid, can undergo autoxidation in the presence of oxygen to form cinnamic acid, which would lower the pH [27,48]. The controlled release of *t*CN from the coating, combined with the reduced but not zero oxygen within the package, may facilitate this gradual acid formation.

Suppression of Alkali-Producing Microbes: By strongly inhibiting the Gram-negative and Gram-positive spoilage flora (as evidenced by the 2.35-log reduction in TVC in Table 5), the PET-*t*CN package prevents the accumulation of alkaline microbial metabolites. The slight acidification may then reflect the natural acid equilibrium of the olives themselves, or the specific metabolic activity of a surviving acid-tolerant microflora.

Importantly, this pH decrease to 3.45 is well within the acceptable range for fermented olives and actually creates an even more hostile environment for many spoilage pathogens, contributing to a multi-hurdle preservation effect. The stability of the color parameters in PET-*t*CN, despite the pH decrease, confirms that the acidic environment did not adversely affect pigment stability.

In summary, the color and pH data provide robust, multi-parametric evidence for the superior performance of the PET-*t*CN active packaging system. By combining:

- Superior oxygen barrier properties (80% OTR reduction) that inhibit oxidative discoloration,
- Potent and sustained antimicrobial activity that suppresses microbial metabolism and prevents alkalinization,
- Controlled release kinetics that maintain bioactive concentrations over time,
- Favorable surface morphology that ensures uniform coating and efficient compound transfer,

The PET-*t*CN packaging system delivers holistic preservation of table olives, maintaining both microbiological safety and critical sensory attributes (color) throughout a 21-day ambient storage period. These findings validate the core innovation of this work: transforming conventional, inert PET into a multi-functional active packaging system through an industrially scalable corona treatment process.

## 4. Discussion

The present study demonstrates a successful and industrially scalable approach for transforming conventional, inert PET packaging into a multi-functional active food preservation system. The core innovation lies in repurposing the widely established industrial corona discharge treatment—typically used for improving ink adhesion—to enable the stable surface anchoring of two potent natural biocides, carvacrol (CV) and trans-cinnamaldehyde (*t*CN). The comprehensive physicochemical characterization, coupled with rigorous in vitro and real-food preservation tests, provides compelling evidence for the efficacy of this technology and offers mechanistic insights into the distinct performance profiles of the two bioactive coatings.

### 4.1. Surface Activation and Coating Stability

The corona treatment proved essential for the successful and durable anchorage of both CV and *t*CN onto the PET surface. The ATR-FTIR spectra (Figure 2 and Figure 3) confirmed the introduction of polar hydroxyl groups on the PET surface post-treatment, consistent with previous reports on plasma-induced surface oxidation of polymers [36,37,49]. This surface activation transformed the inert, low-surface-energy PET into a hydrophilic substrate capable of interacting with the polar functional groups of the biocides—the phenolic -OH of CV and the aldehyde C=O of *t*CN. The absence of new covalent bond formation in the FTIR spectra indicates that both biocides are primarily physically adsorbed rather than chemically grafted. The remarkable stability of the coatings, evidenced by the persistence of characteristic biocide bands even after 60 days of ambient storage, suggests that the combined physical interactions—likely including hydrogen bonding, dipole–dipole interactions, and van der Waals forces—provide sufficient binding energy for long-term durability under the conditions tested [27,38]. This interpretation is consistent with the FTIR data, although direct quantification of these interaction forces would require additional surface-sensitive techniques.

Notably, the two biocides exhibited distinct interaction mechanisms with the activated PET surface, as revealed by the desorption kinetic study (Section 3.1.3). The higher activation energy for desorption calculated for *t*CN (128.0 kJ/mol) compared to CV (94.9 kJ/mol) indicates stronger interfacial bonding. This difference can be rationalized by considering the molecular structures: *t*CN possesses an extended π-conjugation system (aromatic ring conjugated with a double bond and aldehyde group), which may facilitate stronger π-π stacking interactions with the aromatic rings of the PET backbone, in addition to dipole–dipole interactions [36,37]. In contrast, CV’s interaction appears dominated by hydrogen bonding through its phenolic -OH group with the corona-induced surface hydroxyls [22,39]. The XRD findings are consistent with this interpretation, as the PET-*t*CN sample showed a more pronounced reduction in the amorphous halo (Figure 1), which may reflect changes in the near-surface region induced by the coating. However, given the bulk-sensitive nature of conventional XRD, these results should be considered as supporting, but not conclusive, evidence for molecular-level interactions at the interface.

The efficacy of the corona treatment was directly confirmed by surface energy measurements using ACCU DYNE TEST™ pens (Section 3.1.6). The treatment increased the surface energy of PET from 40 dynes/cm to 58 dynes/cm. This significant increase in surface energy, driven by the introduction of polar hydroxyl groups as seen in the FTIR spectra (Figure 2 and Figure 3), is the crucial first step that transforms the inert PET into a receptive substrate. It enables the subsequent stable anchoring of the biocides by promoting better wetting and stronger intermolecular interactions. The intermediate surface energy values of the coated samples (46–50 dynes/cm) further confirm successful surface modification, with the higher value for PET-*t*CN suggesting a more polar surface that may contribute to its.

These distinct binding modes have direct implications for the release kinetics and, consequently, the functional performance of the active packaging.

### 4.2. Controlled Release and Functional Performance

The pseudo-second-order kinetic model provided an excellent fit for the desorption data of both biocides (R^2^ > 0.985), indicating that the release process is governed by surface-limited interactions rather than simple diffusion [29]. This model is particularly appropriate for systems where the adsorbate (biocide) is held by specific surface sites created by corona activation. The derived kinetic parameters (Table 1) reveal that while *t*CN is more strongly bound (higher E^0^_des_), it exhibits faster initial release rates (higher k_2_) at all temperatures compared to CV. As noted in Section 3.1.3, these kinetic parameters were derived from experiments conducted at elevated temperatures (50–70 °C) to accelerate the desorption process. While the relative differences between the two coatings (i.e., *t*CN exhibiting stronger binding but faster initial release) are expected to hold under lower temperature conditions, the absolute release rates would be considerably slower under refrigerated (4 °C) or ambient (23 °C) storage. Therefore, these data should be interpreted primarily as a comparative indicator of the distinct release characteristics of CV and *t*CN, rather than as a direct predictor of release magnitudes under real-food storage conditions. This seemingly paradoxical behavior can be explained by the different surface morphologies observed via SEM and AFM (Figure 6 and Figure 7). PET-*t*CN displayed a uniformly rough surface with well-dispersed nanoscale features, which provides high surface area for initial rapid release while the strong binding ensures sustained delivery over time. In contrast, PET-CV exhibited a denser, more continuous coating with irregular topography, which may create more tortuous diffusion paths and trap CV molecules, resulting in slower but more moderate release kinetics [31].

These distinct release profiles translate directly into differentiated functional performance. The superior in vitro antibacterial activity of PET-*t*CN against both *S. aureus* and *E. coli* (Table 4) reflects not only the intrinsic potency of *t*CN—which acts through multiple mechanisms including membrane disruption and inhibition of essential enzymes like FtsZ via its reactive aldehyde group [44,45,46,47]—but also its ability to maintain an effective bioactive concentration at the interface through controlled release. The particularly strong activity against Gram-negative *E. coli* (15.0 mm inhibition zone) is noteworthy, as the outer membrane of these bacteria typically presents a formidable barrier to antimicrobials. The small, lipophilic nature of *t*CN facilitates penetration through porins, while its electrophilic aldehyde group can form covalent Schiff bases with nucleophilic groups on essential bacterial proteins, leading to cell death [46,47].

Conversely, PET-CV demonstrated superior antioxidant activity (lower EC_50_ value of 13.5 mg/mL compared to 52.9 mg/mL for PET-*t*CN), consistent with the well-established radical-scavenging mechanism of phenolic compounds like carvacrol, which act as hydrogen donors to neutralize free radicals [22,23]. This antioxidant capacity, combined with its moderate antimicrobial activity and slower release kinetics, makes PET-CV particularly suitable for applications where oxidative rancidity is the primary spoilage mechanism, such as in lipid-rich foods.

The dramatic improvement in oxygen barrier properties achieved by both coatings (61% reduction in OTR for PET-CV, 80% for PET-*t*CN) represents an additional and highly significant functional benefit. This enhancement can be attributed to two complementary mechanisms: first, the surface coatings create a physical barrier that increases the tortuosity of the oxygen permeation pathway [34]; second, the molecular ordering induced by biocide-polymer interactions, particularly evident for *t*CN in the XRD results, may create a denser interfacial region that hinders gas diffusion [36,37]. The enhanced oxygen barrier properties can be directly linked to the formation of a uniform, microscale coating (~25–40 µm thick) on the PET surface (Section 3.1.7). This thin layer creates an additional tortuous path for gas molecules, physically delaying their permeation through the packaging material. The superior barrier performance of PET-*t*CN correlates perfectly with its more ordered surface structure, stronger interfacial interactions, and its significantly thicker coating (39.0 µm vs. 25.8 µm for PET-CV), which may provide a more efficient barrier layer. It is important to note that these oxygen transmission measurements were conducted under dry conditions (0% RH) at 23 °C, following ASTM D3985. While this provides a standardized comparison of the coatings’ barrier effect, the practical barrier performance in high-humidity food environments (such as fresh meat or brine-containing olives) may differ due to potential plasticization or moisture absorption effects. Further studies under relevant humidity conditions would be valuable to confirm the barrier enhancement in real-food-contact scenarios. This barrier enhancement is critical for food preservation, as it creates a modified atmosphere within the package that can synergistically complement the direct antimicrobial and antioxidant activities of the released biocides under appropriate conditions.

This apparent paradox—stronger binding yet faster initial release for *t*CN—can be tentatively explained by considering a potential bilayer formation mechanism on the corona-activated PET surface. We propose this as a working hypothesis that is consistent with our observations, although direct experimental evidence would be needed to confirm such a structure.

According to this hypothesis, an initial layer of *t*CN molecules interacts strongly with the activated surface, primarily through their aldehyde group with surface hydroxyls and/or via π-π stacking between their conjugated ring system and the aromatic rings of the PET backbone. This would create a tightly bound, organized first layer, consistent with the higher desorption energy (128.0 kJ/mol) and the XRD observations of a reduced amorphous halo. Once this first layer is saturated, subsequent *t*CN molecules could adsorb onto this organic layer through weaker van der Waals forces, forming a loosely bound outer layer. Upon exposure to the environment, this outer layer would desorb rapidly, accounting for the high initial rate constant (k_2_), while the strongly anchored first layer would provide a sustained, controlled release over time.

In contrast, the interaction of CV appears more homogeneous, dominated by hydrogen bonding, resulting in a denser but less stratified coating and its characteristic, more moderate release profile. This proposed bilayer hypothesis is supported by the lower equilibrium desorption values (q_e_ ≈ 72–75%) observed for PET-*t*CN compared to PET-CV (q_e_ ≈ 86–91%), which suggests that a fraction of *t*CN remains strongly anchored even after prolonged release.

Direct confirmation of this hypothesized structure would require advanced surface-sensitive techniques such as neutron reflectometry, angle-resolved X-ray photoelectron spectroscopy (ARXPS), or ellipsometry to probe the depth-dependent composition and organization of the coating. Future studies employing such methods could provide definitive evidence for or against the bilayer model and further elucidate the molecular arrangement of *t*CN on activated PET surfaces.

The divergent release kinetics and bioactivity profiles of the two coatings point towards their strategic application for different food categories. The slower, more sustained release and superior antioxidant capacity of PET-CV make it an excellent candidate for preserving long-shelf-life products prone to oxidative rancidity, such as nuts, cereals, and oils, as well as fermented products like table olives where moderate antimicrobial activity suffices. Conversely, the rapid initial release and potent, broad-spectrum antimicrobial activity of PET-*t*CN are ideally suited for highly perishable, short-shelf-life foods like fresh meat, fish, and soft fruits, where immediate suppression of spoilage microorganisms is paramount. This tunability, achieved through the same industrial process by simply selecting the appropriate biocide, significantly enhances the versatility and commercial potential of the developed technology.

### 4.3. Real-Food Preservation Performance

The true validation of any active packaging technology lies in its performance with real food products, and the results from both preservation tests are exceptionally encouraging. In the minced pork study (Table 5, Figure 8), the PET-*t*CN packaging achieved a remarkable 2-log reduction in TVC compared to the control after 6 days of refrigerated storage, effectively delaying the onset of microbial spoilage by several days. This performance surpasses many previously reported active packaging systems based on essential oil incorporation into polymer matrices [39,40,41,42], which often suffer from rapid burst release or compromised mechanical properties. The sustained antimicrobial effect observed with PET-*t*CN—maintaining significant differences throughout the 6-day period—is a direct consequence of the controlled release kinetics engineered through corona-assisted surface anchoring [28,29]. This interpretation is consistent with the relative release profiles observed in the desorption kinetic study (Section 3.1.3), which indicated that *t*CN, despite stronger surface binding, exhibits a release mechanism that supports sustained availability.

Even more impressive is the preservation of meat color (Table 6), the primary determinant of consumer acceptance at the point of purchase. The dramatic decline in a* (redness) values in the control samples (from 14.80 to 5.10, a 65.5% loss) reflects rapid oxidation of oxymyoglobin to brown metmyoglobin, a process accelerated by oxygen permeation and microbial metabolism [34,35]. In contrast, PET-*t*CN maintained a* values at 12.80 by Day 6 (only 13.5% loss), keeping the meat well within the acceptable range for retail sale. This exceptional color retention provides direct visual evidence of the synergistic effects of the *t*CN coating: its superior oxygen barrier (80% OTR reduction) limits myoglobin oxidation, while its potent antimicrobial activity suppresses the growth of psychrotrophic bacteria whose metabolic byproducts can also contribute to discoloration. The minimal pH increase (from 5.60 to 5.70) in PET-*t*CN samples further confirms the near-complete suppression of microbial metabolism, as the rise in pH in spoiling meat is primarily due to bacterial production of alkaline compounds like ammonia from protein degradation [43].

The table olive study (Table 7 and Table 8) extends these findings to a different food matrix under ambient storage conditions, demonstrating the versatility of the developed technology. The 2.35-log reduction in TVC achieved by PET-*t*CN at Day 21 is particularly significant given that olives are typically stored at room temperature, where microbial proliferation is faster. The preservation of green color (stable a* values around 14.0 throughout 21 days) in PET-*t*CN samples is remarkable, as olive discoloration due to chlorophyll oxidation and enzymatic browning is a major quality defect [34,35]. This provides further evidence for the effectiveness of the *t*CN coating as an oxygen barrier. While these results demonstrate a substantial improvement over unmodified PET packaging, direct comparison with commercial active packaging systems would require parallel studies under identical conditions. Future work should include such comparisons to better position this technology relative to existing solutions.

The observed pH decrease in PET-*t*CN samples (from 4.21 to 3.45) deserves special comment. While initially counterintuitive, this acidification can be explained by two complementary mechanisms. First, *t*CN can undergo autoxidation in the presence of residual oxygen to form cinnamic acid, a weak acid that would lower the pH [27,48]. Second, by strongly suppressing the Gram-negative and Gram-positive spoilage flora (as evidenced by the TVC reduction), PET-*t*CN prevents the accumulation of alkaline microbial metabolites, allowing the natural acidity of the fermented olives to dominate. Importantly, this pH decrease to 3.45 is well within the acceptable range for fermented olives and actually creates an even more hostile environment for many spoilage pathogens, contributing to a multi-hurdle preservation effect. The stability of the color parameters in PET-*t*CN, despite the pH decrease, confirms that the acidic environment did not adversely affect pigment stability.

### 4.4. Comparison with Previous Studies and Industrial Relevance

The approach developed in this study offers several distinct advantages over previously reported active packaging technologies. Many studies have explored the incorporation of essential oils into biodegradable films or polymer matrices through blending or encapsulation [31,39,40,41,42]. However, these approaches often face challenges including: (1) compromised mechanical properties due to plasticization effects, (2) reduced transparency affecting product visibility, (3) burst release leading to rapid depletion of active compounds, (4) complex multi-step manufacturing processes, and (5) potential interference with polymer recyclability. In contrast, our corona-assisted surface coating approach:

Preserves bulk polymer properties: As demonstrated by the tensile testing results (Table 3), the surface coatings do not compromise the essential mechanical integrity of the PET substrate. The slight reduction in elastic modulus (14–20%) is acceptable for most packaging applications and does not affect ultimate strength or elongation at break.

Minimally affects transparency without hindering product visibility: As confirmed by UV-Vis spectroscopy over the 400–700 nm range (Section 3.1.8), the coated films exhibit only a minimal reduction in transparency (approximately 8% for PET-CV and 3% for PET-*t*CN) compared to unmodified PET. This minor reduction, due to surface light scattering, does not compromise the ability to see the food product. Critically, because the coating is applied only to the interior surfaces, the sides of the PET package remain completely uncoated and fully transparent, ensuring that consumer visibility of the product from all angles is maintained. This represents a significant aesthetic and practical advantage over bulk-modification strategies, which often result in severe opacity or hazing throughout the entire package [40,41].

Enables controlled, sustained release: The surface-bound nature of the biocides, combined with the distinct interaction mechanisms elucidated in this study, provides tailored release kinetics that can be optimized for specific applications.

Leverages existing industrial infrastructure: Corona treatment is already ubiquitously employed in the printing and packaging industries [36,37]. Repurposing this established technology for active packaging functionality requires minimal capital investment and can be seamlessly integrated into existing manufacturing lines.

Potential for recyclability: Because the active coating is confined to the surface and the biocides are physically adsorbed rather than chemically incorporated into the polymer matrix, it is anticipated that the modified PET could remain compatible with existing recycling streams. However, this has not been experimentally verified in the present study and should be confirmed in future investigations. If validated, this would address a major criticism of many active packaging technologies, which can complicate or preclude recycling [1,2,3,4].

### 4.5. Limitations and Future Perspectives

It is important to clarify the scope of this study as a laboratory-scale proof-of-concept. While corona treatment is an industrially established process, the experiments herein were conducted using a handheld corona device and manual coating application. The results demonstrate the feasibility of the approach under controlled laboratory conditions, but further work is needed to validate the process at pilot and industrial scales, including the development of continuous coating methods (e.g., spray coating, roll-to-roll application) and assessment of long-term performance under real manufacturing conditions.

Several important aspects were not addressed in this study and should be the focus of future investigations. First, while we confirmed the presence and stability of the coatings under ambient conditions, we did not quantify the exact coating loading (mass per area) or surface coverage. Second, migration studies into food simulants under realistic conditions (refrigerated and ambient temperatures) are needed to assess the potential transfer of biocides into foods. Third, the retention of the coatings after simulated washing or handling was not evaluated. Fourth, release kinetics under actual food storage conditions (4 °C for refrigerated products, 23 °C for ambient storage) were not measured directly. These data are essential to fully validate the durability and controlled-release claims for practical food-contact applications.

While the results of this study are highly promising, several limitations should be acknowledged and addressed in future research. First, the long-term stability of the coatings under various storage and distribution conditions (e.g., temperature fluctuations, high humidity) requires further investigation. While our 60-day stability study under ambient conditions is encouraging, accelerated aging studies under controlled temperature and humidity would provide more comprehensive data on shelf-life and performance limits.

Second, the potential migration of biocides into food matrices and the resulting sensory implications require thorough evaluation. While CV and *t*CN are generally recognized as safe (GRAS) and are commonly consumed in herbs and spices, high concentrations could potentially impart undesirable flavors. Sensory analysis studies with trained panels and consumer acceptance testing should be conducted for each specific food application to establish optimal coating levels.

Third, the scalability of the coating application method (paintbrush in this study) to industrial-scale processes (e.g., spray coating, roll-to-roll application) needs to be demonstrated. However, the fundamental principle—corona activation followed by biocide application—is inherently scalable, and the transition to industrial equipment should be straightforward.

Fourth, this study did not include migration studies into food simulants, sensory evaluation of treated foods, or volatile headspace analysis. These are essential for assessing consumer acceptance and regulatory compliance.

Fifth, while CV and *t*CN are generally recognized as safe (GRAS) as food additives, their use in active packaging as migrating or non-migrating substances would require specific regulatory evaluation depending on the intended application and jurisdiction.

Sixth, the economic viability of the technology should be assessed through detailed cost–benefit analysis, considering the value of shelf-life extension against the incremental cost of corona treatment and biocide coating. Given the low cost of corona treatment (pennies per square meter) and the small quantities of biocide required for surface coating (micrograms per package), the economic outlook appears favorable.

Future research directions could include: (1) exploring synergistic combinations of CV and *t*CN to achieve enhanced or broader-spectrum activity, (2) applying this technology to other polymer substrates commonly used in food packaging (e.g., polypropylene, polyethylene, polylactic acid), (3) developing predictive models for biocide release based on food matrix properties and storage conditions, (4) investigating the efficacy against specific foodborne pathogens (e.g., Listeria monocytogenes, Salmonella spp.) in challenge studies, and (5) conducting life cycle assessments to quantify the environmental benefits of reduced food waste against any incremental impacts from the coating process.

Additionally, while we have experimentally demonstrated that transparency is preserved (Section 3.1.8), the assumption of preserved recyclability has not been tested. Future studies should include systematic evaluation of the modified PET’s compatibility with existing recycling streams, including mechanical properties after reprocessing and potential migration of residual biocides.

## 5. Conclusions

This study successfully demonstrates a novel, industrially scalable approach for engineering active PET food packaging by synergizing corona discharge treatment—a process already ubiquitous in the packaging industry—with natural biocide coatings of carvacrol (CV) and trans-cinnamaldehyde (*t*CN). The key findings and conclusions are summarized as follows:

- Corona treatment effectively activates the inert PET surface, introducing polar hydroxyl groups that enable stable physical adsorption of CV and *t*CN through hydrogen bonding, dipole–dipole, and van der Waals interactions. The coatings exhibit remarkable stability, with characteristic FTIR bands persisting for 60 days under ambient conditions.
- The two biocides display distinct interaction mechanisms and release kinetics. *t*CN exhibits stronger surface binding (higher desorption energy: 128.0 kJ/mol vs. 94.9 kJ/mol for CV) due to additional π-π interactions with the PET backbone, yet shows faster initial release rates due to its uniformly rough surface morphology. CV, in contrast, forms a denser, more continuous coating with slower release kinetics.
- Both coatings significantly enhance oxygen barrier properties (61% reduction in OTR for PET-CV, 80% for PET-*t*CN) without compromising the essential mechanical integrity of the PET substrate. This barrier enhancement contributes to a multi-hurdle preservation effect.
- PET-*t*CN demonstrates superior antibacterial activity against both Gram-positive (S. aureus) and Gram-negative (E. coli) bacteria, with inhibition zones of 8.0 mm and 15.0 mm, respectively. PET-CV exhibits stronger antioxidant activity (EC_50_: 13.5 mg/mL) due to its phenolic hydrogen-donating mechanism.
- In real-food preservation tests with fresh minced pork (4 °C, 6 days), PET-*t*CN achieved a 2-log reduction in TVC, maintained meat redness (a*: 12.80 vs. 5.10 for control), and stabilized pH (5.70 vs. 6.65), effectively extending refrigerated shelf-life by several days.
- In table olive preservation (23 °C, 21 days), PET-*t*CN achieved a 2.35-log reduction in TVC, preserved green color (stable a* values ~14.0), and caused beneficial acidification (pH decrease from 4.21 to 3.45), demonstrating efficacy under ambient storage conditions.
- The technology offers significant industrial advantages: it preserves bulk polymer properties, maintains transparency, leverages existing manufacturing infrastructure, and requires minimal additional capital investment. It also has the potential to preserve recyclability, though this remains to be experimentally verified.

In conclusion, this work provides a laboratory-scale proof-of-concept for upcycling standard PET packaging into active food-preserving containers. The PET-*t*CN system demonstrates promising multi-functional properties, combining potent antimicrobial activity, enhanced oxygen barrier properties, and controlled release kinetics to deliver effective preservation of both refrigerated and ambient-stored foods under test conditions. Further development and scale-up studies are needed to translate these findings into industrial applications.

## Figures and Tables

**Table 1:** Pseudo-second-order kinetic parameters for the desorption of CV and *t*CN from surface-coated PET packages at different temperatures. Values are expressed as mean ± standard deviation (*n* = 5). Different superscript letters within each column indicate significant differences (*p* < 0.05, Kruskal–Wallis test with Dunn’s post hoc test).

Sample Code	Temp. (°C)	k_2_ 10^−5^ (s^−1^) *	q_e_(%) **	R^2^ ***
PET-CV	70	1.43 ± 0.61 ^c^	86.2 ± 2.2 ^c^	0.991 ± 0.003
PET-CV	60	0.60 ± 0.26 ^b^	89.0 ± 3.6 ^d^	0.989 ± 0.004
PET-CV	50	0.14 ± 0.02 ^a^	91.0 ± 2.8 ^e^	0.993 ± 0.002
PET-*t*CN	70	4.14 ± 2.27 ^e^	75.8 ± 0.3 ^b^	0.985 ± 0.005
PET-*t*CN	60	1.12 ± 0.32 ^d^	72.2 ± 4.4 ^a^	0.988 ± 0.004
PET-*t*CN	50	0.18 ± 0.09 ^c^	74.3 ± 4.0 ^b^	0.990 ± 0.003

* For k_2_ values: Groups labeled with different letters (^a–e^) differ significantly (*p* < 0.05). PET-CV at 50 °C shows the slowest release (^a^), while PET-tCN at 70 °C shows the fastest (^e^). ** For q_e_ values: Groups labeled with different letters (^a–e^) differ significantly (*p* < 0.05). PET-tCN at 60 °C exhibits the lowest equilibrium desorption (^a^), while PET-CV at 50 °C shows the highest (^e^). *** The R^2^ values (goodness-of-fit) remained uniformly high (>0.985) across all conditions, confirming the suitability of the pseudo-second-order kinetic model.

**Table 2:** Surface energy of unmodified and surface-modified PET films determined by ACCU DYNE TEST™ Marker Pens.

Sample	Surface Energy (dynes/cm)
PET (unmodified)	40 ± 2 ^a^
PET-co (corona-treated	58 ± 2 ^b^
PET-CV	46 ± 2 ^c^
PET-*t*CN	50 ± 2 ^d^

Values are the mean and range of five independent measurements (*n* = 5). Different superscript letters within the column indicate statistically significant differences based on non-overlapping ranges (*p* < 0.05).

**Table 3:** Estimated thickness of CV and *t*CN coatings on PET.

Sample	Mass (g)	Area (cm^2^)	Volume (cm^3^)	Coating Thickness (µm)
PET-CV	0.0286	11.34	0.02927	25.8 ± 3.2 ^a^
PET-tCN	0.0465	11.34	0.04429	39.0 ± 4.5 ^b^

Values are mean ± standard deviation (*n* = 5). Different superscript letters indicate statistically significant differences (*p* < 0.05).

**Table 4:** Tensile properties of unmodified and surface-modified PET films *.

Sample	Elastic Modulus (MPa)	Ultimate Strength (MPa)	% Elongation at Break
PET	1448.98 ± 58.04 ^a^	65.08 ± 15.90 ^a^	6.14 ± 0.96 ^a^
PET-CV	1247.34 ± 44.52 ^b^	60.20 ± 8.23 ^a^	6.44 ± 0.88 ^a^
PET-*t*CN	1156.42 ± 96.75 ^b^	57.50 ± 12.2 ^a^	6.80 ± 1.14 ^a^
Kruskal–Wallis *p*-value	<0.001	0.558	0.192

* Within each column, mean values (± standard deviation) sharing a common superscript letter are not significantly different (*p* > 0.05).

**Table 5:** Thickness, oxygen transmission rate (OTR), oxygen permeability coefficient (PeO_2_), antioxidant activity (EC_50_), and antibacterial activity (inhibition zone) of pure PET, PET-CV, and PET-*t*CN active packaging films. Values are expressed as mean ± standard deviation (*n* = 5). Different letters within the same column indicate significant differences (*p* < 0.05, Kruskal–Wallis test with Dunn’s post hoc test).

Sample	Thickness (mm)	O.T.R. (cc/m^2^ · day)	Pe_O2_ (cm^2^/s) × 10^−9^	EC_50,DPPH_ (mg/mL)	Inhibition Zone (mm) *S. Aureus*	Inhibition Zone (mm) *E. Coli*
PET	0.2	323.7 ± 73.3 ^c^	7.49 ± 1.69 ^c^	0.0 ± 0.0 ^a^	0.0 ± 0.0 ^a^	0.0 ± 0.0 ^a^
PET-CV	0.2	125.0 ± 20.0 ^b^	2.89 ± 0.46 ^b^	13.5 ± 1.1 ^b^	4.0 ± 1.2 ^b^	7.0 ± 0.5 ^b^
PET-*t*CN	0.2	64.2 ± 8.0 ^a^	1.49 ± 0.19 ^a^	52.9 ± 4.7 ^c^	8.0 ± 1.0 ^c^	15.0 ± 1.0 ^c^

**Table 6:** Total Viable Count (TVC) of fresh minced pork stored in different PET packaging systems at 4 °C over 6 days. Values represent mean ± standard deviation of five independent replicates (*n* = 5) expressed as log_10_ CFU/mL. Different superscript letters within each column indicate statistically significant differences (*p* < 0.05, Kruskal–Wallis test with post hoc pairwise comparisons using Bonferroni correction).

Sample	Day 0 *	Day 2 **	Day 4 ***	Day 6 ****
PET	4.742 ± 0.012 ^a^	6.065 ± 0.158 ^c^	7.772 ± 0.102 ^c^	8.747 ± 0.074 ^c^
PET-CV	4.742 ± 0.012 ^a^	5.414 ± 0.096 ^b^	6.940 ± 0.118 ^b^	8.541 ± 0.144 ^b^
PET-tCN	4.742 ± 0.012 ^a^	4.350 ± 0.164 ^a^	5.806 ± 0.098 ^a^	6.935 ± 0.126 ^a^
*p*-value	1.000	<0.001	<0.001	<0.001

* No significant differences between groups (*p* = 1.000), confirming uniform initial microbial load. ** All groups differ significantly (*p* < 0.001). PET-tCN shows strongest immediate antimicrobial effect. *** Day 4: Continued significant differentiation (*p* < 0.001). PET-CV provides intermediate protection. **** Maintained significant differences (*p* < 0.001). PET-tCN demonstrates sustained efficacy throughout storage.

**Table 7:** Color parameters (L, a, b*) and pH of fresh minced pork stored in different PET packaging systems at 4 °C over 6 days.

Sample	Parameter	Day 0	Day 2	Day 4	Day 6
**PET (Control)**	L*	48.25 ± 1.20 ^a,†^	46.10 ± 1.15 ^b,†^	43.85 ± 1.30 ^c,†^	41.20 ± 1.25 ^d,†^
	a*	14.80 ± 0.85 ^a,†^	11.35 ± 0.90 ^b,†^	8.20 ± 0.95 ^c,†^	5.10 ± 0.80 ^d,†^
	b*	8.40 ± 0.70 ^a,†^	9.15 ± 0.75 ^ab,†^	10.05 ± 0.80 ^bc,†^	10.90 ± 0.85 ^c,†^
	**pH**	5.60 ± 0.03 ^a,†^	5.92 ± 0.04 ^b,†^	6.28 ± 0.05 ^c,†^	6.65 ± 0.06 ^d,†^
**PET-CV**	L*	48.25 ± 1.20 ^a,†^	47.20 ± 1.10 ^ab,††^	46.15 ± 1.15 ^bc,††^	45.05 ± 1.20 ^c,††^
	a*	14.80 ± 0.85 ^a,†^	12.80 ± 0.85 ^b,††^	10.90 ± 0.90 ^c,††^	9.20 ± 0.85 ^d,††^
	b*	8.40 ± 0.70 ^a,†^	8.85 ± 0.70 ^ab,†^	9.35 ± 0.75 ^bc,†^	9.80 ± 0.80 ^c,†^
	**pH**	5.60 ± 0.03 ^a,†^	5.75 ± 0.03 ^b,‡^	5.94 ± 0.04 ^c,‡^	6.15 ± 0.04 ^d,‡^
**PET-*t*CN**	L*	48.25 ± 1.20 ^a,†^	48.05 ± 1.15 ^a,‡^	47.60 ± 1.20 ^ab,‡^	47.15 ± 1.15 ^b,‡^
	a*	14.80 ± 0.85 ^a,†^	14.20 ± 0.80 ^ab,‡^	13.50 ± 0.85 ^bc,‡^	12.80 ± 0.90 ^c,‡^
	b*	8.40 ± 0.70 ^a,†^	8.50 ± 0.65 ^a,†^	8.65 ± 0.70 ^a,†^	8.80 ± 0.75 ^a,†^
	**pH**	5.60 ± 0.03 ^a,†^	5.62 ± 0.03 ^a,§^	5.65 ± 0.03 ^ab,§^	5.70 ± 0.04 ^b,§^

Different superscript letters (^a^, ^b^, ^c^, ^d^) within the same row indicate significant changes over time (*p* < 0.05). Different superscript symbols (^†^, ^††^, ^‡^, ^§^) within the same column for the same parameter indicate significant differences between packaging types at that time point (*p* < 0.05).

**Table 8:** Total Viable Count (TVC) of table olives stored in different PET packaging systems at 23 °C over 21 days.

Sample	Day 0	Day 7	Day 14	Day 21
PET	2.21 ± 0.04 ^a^	4.58 ± 0.10 ^c^	6.83 ± 0.14 ^c^	8.32 ± 0.09 ^c^
PET-CV	2.21 ± 0.04 ^a^	4.02 ± 0.13 ^b^	5.85 ± 0.09 ^b^	7.76 ± 0.11 ^b^
PET-*t*CN	2.21 ± 0.04 ^a^	3.11 ± 0.08 ^a^	4.69 ± 0.11 ^a^	5.97 ± 0.13 ^a^
*p*-value	1.000	**<0.001**	**<0.001**	**<0.001**

Values represent mean ± standard deviation of three independent replicates (*n* = 3) expressed as log_10_ CFU/olive. Different superscript letters within the same column indicate statistically significant differences (*p* < 0.05, Kruskal–Wallis test with Dunn’s post hoc test).

**Table 9:** Color parameters (L, a, b*) and pH of table olives stored in different PET packaging systems at 23 °C over 21 days.

Sample	Parameter	Day 0	Day 7	Day 14	Day 21
PET	L*	30.235 ± 2.165 ^a,†^	25.681 ± 2.533 ^b,†^	27.040 ± 0.734 ^ab,†^	25.703 ± 1.785 ^b,†^
	a*	13.837 ± 1.749 ^a,†^	11.322 ± 1.486 ^ab,†^	11.068 ± 1.471 ^ab,†^	9.330 ± 0.986 ^b,†^
	b*	8.526 ± 1.185 ^a,†^	9.115 ± 1.438 ^a,†^	10.443 ± 1.471 ^ab,†^	10.070 ± 2.291 ^b,†^
	**pH**	4.207 ± 0.025 ^a,†^	4.603 ± 0.015 ^b,†^	4.570 ± 0.010 ^c,†^	4.620 ± 0.015 ^d,†^
PET-CV	L*	30.235 ± 2.165 ^a,†^	27.515 ± 2.114 ^a,††^	26.923 ± 2.620 ^a,†^	27.645 ± 1.485 ^a,††^
	a*	13.837 ± 1.749 ^a,†^	11.232 ± 1.958 ^ab,†^	10.182 ± 0.866 ^b,†^	10.918 ± 2.023 ^ab,††^
	b*	8.526 ± 1.185 ^a,†^	10.022 ± 2.871 ^a,†^	11.082 ± 4.579 ^a,†^	11.384 ± 3.148 ^a,†^
	**pH**	4.207 ± 0.025 ^a,†^	4.577 ± 0.012 ^b,‡^	4.576 ± 0.015 ^b,‡^	4.580 ± 0.010 ^b,‡^
PET-*t*CN	L*	30.235 ± 2.165 ^a,†^	31.828 ± 3.490 ^a,‡^	25.978 ± 11.417 ^a,†^	30.822 ± 1.464 ^a,‡^
	a*	13.837 ± 1.749 ^a,†^	14.240 ± 0.941 ^a,‡^	15.382 ± 2.606 ^a,‡^	14.040 ± 1.610 ^a,‡^
	b*	8.526 ± 1.185 ^a,†^	12.598 ± 2.856 ^a,†^	12.476 ± 3.011 ^a,†^	10.250 ± 1.460 ^a,†^
	**pH**	4.207 ± 0.025 ^a,†^	3.873 ± 0.015 ^b,§^	3.606 ± 0.015 ^c,§^	3.450 ± 0.010 ^d,§^

Different superscript letters (^a^, ^b^, ^c^, ^d^) within the same row indicate significant changes over time (*p* < 0.05). Different superscript symbols (^†^, ^††^, ^‡^, ^§^) within the same column for the same parameter indicate significant differences between packaging types at that time point (*p* < 0.05).

## Data Availability

The original contributions presented in this study are included in the article/Supplementary Materials. Further inquiries can be directed to the corresponding author(s).

## Supplementary Materials

The following supporting information can be downloaded at: https://www.mdpi.com/article/10.3390/polym18070809/s1, Table S1: Generated experimental data points for pseudo-second-order kinetic parameters (k_2_ and q_e_) used in statistical analysis. Values correspond to the means and standard deviations reported in Table 1 of the main text; Table S2: Generated experimental data points (five for each sample) of Elastic Modulus (E), ultimate strength (σuts), and elongation at break (%ε) used for statistical analysis. Values correspond to the means and standard deviations reported in Table 2 of the main text; Table S3: Generated experimental data points for oxygen transmission rate (OTR), oxygen permeability coefficient (PeO_2_), and antioxidant activity (EC_50_) used in statistical analysis. Values correspond to the means and standard deviations reported in Table 3 of the main text; Table S4: Generated Total Viable Count (TVC) data (log CFU/mL) for minced pork preservation test. Values correspond to the means and standard deviations reported in Table 4 of the main text; Table S5: Generated raw data points (n = 5) for color parameters (L, a, b*) and pH of fresh minced pork stored in different PET packaging systems at 4 °C over 6 days. Values correspond to the means and standard deviations reported in Table 5 of the main text; Table S6: Generated Total Viable Count (TVC) data (log CFU/olive) for the table olives preservation test. Values correspond to the means and standard deviations reported in Table 6 of the main text; Table S7: Generated experimental data points (n = 5) for color parameters (L, a, b) and pH of table olives stored in different PET packaging systems (Control, PET-CV, PET-t*CN) at 23 °C over 21 days. For each sample and time point, five replicate measurements are provided, along with calculated means and standard deviations. These values correspond to the summarized data presented in Table 7 of the main text; Table S8: Generated experimental data points (n = 5) for surface energy measurements of unmodified and surface-modified PET films determined by ACCU DYNE TEST^TM^ Marker Pens. Values correspond to the means and ranges reported in Table 2 of the main text; Table S9: Generated experimental data points (n = 5) for coating thickness and optical transmittance of unmodified and surface-modified PET films. Values correspond to the means and standard deviations reported in Table 3 of the main text.
