# Supplementary material for: Engineering Active PET Packaging via Corona Treatment and Natural Biocide Coating: Carvacrol and Trans-Cinnamaldehyde for Food Preservation
Source: Polymers (Basel). 2026 Mar 26;18(7):809. doi: 10.3390/polym18070809 (PMC13074524; doi:10.3390/polym18070809)
Supplement: Supplementary file 1 [file polymers-18-00809-s001.zip › polymers-4208198-supplementary.pdf]

Supplementary material file for:

Engineering active PET packaging via corona treatment and natural biocide coating: Carvacrol and *trans*-cinnamaldehyde for food preservation

Pantelis Karaboulis[1], Areti A. Leontiou[1], Christos Tsakonas[2,3], George Paterakis[2,3], Andreas E. Giannakas[1], Panagiota Stathopoulou[4], Charalampos Proestos[5], Costas Galiotis[2,3], Constantinos E. Salmas[6] and Aris E. Giannakas [1,*]

[1] Department of Food Science and Technology, University of Patras, 30100 Agrinio, Greece; up1120174@upatras.gr (P.K.); aleontiu@upatras.gr (A.A.L.); andgiannakas@upatras.gr (A.G.)
[2] Department of Chemical Engineering, University of Patras; 26504 Patras, Greece; ctsako@chemeng.upatras.gr (C.T.)
[3] Institute of Chemical Engineering Sciences, Foundation for Research and Technology – Hellas (FORTH/ ICE-HT), Patras, Greece; gpaterakis@iceht.forth.gr (G.P.); c.galiotis@iceht.forth.gr (C.G.)
[4] Department of Sustainable Agriculture, University of Patras, 30100 Agrinio, Greece; panstath@upatras.gr (P.S.)
[5] Laboratory of Food Chemistry, Department of Chemistry, National and Kapodistrian University of Athens,
Zografou, 15771 Athens, Greece; harpro@chem.uoa.gr (C.P.)
[6] Department of Material Science and Engineering, University of Ioannina, 45110 Ioannina, Greece, ksalmas@uoi.gr (C.E.S.)
* Correspondence: agiannakas@upatras.gr

Table S1. Generated experimental data points for pseudo-second-order kinetic parameters (k_2 and q_e) used in statistical analysis. Values correspond to the means and standard deviations reported in Table 1 of the main text.

Sample Code	Temp. (°C)	Replicate	k_2 ($\times 10^{-5}$ s^{-1})	q_e (%)	R^2
**PET-CV**	70	1	1.05	83.5	0.991
		2	1.20	84.8	0.993
		3	1.43	86.2	0.990
		4	1.66	87.5	0.992
		5	1.81	88.9	0.988
**PET-CV**	60	1	0.34	84.5	0.985
		2	0.45	86.7	0.991
		3	0.60	89.0	0.989
		4	0.75	91.3	0.990
		5	0.86	93.5	0.987
**PET-CV**	50	1	0.12	87.5	0.995
		2	0.13	89.2	0.994
		3	0.14	91.0	0.993
		4	0.15	92.8	0.992
		5	0.16	94.5	0.991
**PET-*t*CN**	70	1	1.87	75.5	0.980

		2	3.00	75.7	0.987
		3	4.14	75.8	0.985
		4	5.27	75.9	0.984
		5	6.40	76.1	0.983
**PET-*t*CN**	60	1	0.80	66.0	0.985
		2	0.96	69.1	0.989
		3	1.12	72.2	0.988
		4	1.28	75.3	0.990
		5	1.44	78.4	0.987
**PET-*t*CN**	50	1	0.09	69.5	0.988
		2	0.13	71.9	0.991
		3	0.18	74.3	0.990

Table S2. Generated experimental data points (five for each sample) of Elastic Modulus (E), ultimate strength (σ_{uts}), and elongation at break (%ε) used for statistical analysis. Values correspond to the means and standard deviations reported in Table 2 of the main text.

	**Elastic Modulus**	**σ_{uts}**	**%ε**
**PET_1**	1475.9	65.9	6.6
**PET_2**	1470.5	66.8	6.5
**PET_3**	1390.6	46.4	5.1
**PET_4**	1520.4	89.4	7.3
**PET_5**	1387.5	56.9	5.2
**PET-CN_1**	1198.7	62.1	7.1
**PET-CN_2**	997.1	48.9	8.1
**PET-CN_3**	1208.2	63.2	6.6
**PET-CN_4**	1136.8	41.3	5
**PET-CN_5**	1241.3	72	7.2
**PET-CV_1**	1201.5	55.5	6.1
**PET-CV_2**	1234.4	68.5	7.7
**PET-CV_3**	1276.8	54.3	5.6
**PET-CV_4**	1215.3	52.9	5.8
**PET-CV_5**	1308.7	69.8	7

Table S3. Generated experimental data points for oxygen transmission rate (OTR), oxygen permeability coefficient (PeO_2), and antioxidant activity (EC_{50}) used in statistical analysis. Values correspond to the means and standard deviations reported in Table 3 of the main text.

**Sample**	**Replicate**	**OTR ($cm^3/m^2 \cdot day$)**	**PeO_2 ($\times 10^{-9}$ cm^2/s)**	**EC_{50} (mg/mL)**
**PET**	1	250.0	5.78	0.0
	2	300.0	6.94	0.0
	3	323.7	7.49	0.0
	4	350.0	8.10	0.0
	5	375.0	8.68	0.0

**PET-CV**	1	105.0	2.43	12.0
	2	115.0	2.66	13.0
	3	125.0	2.89	13.5
	4	135.0	3.12	14.0
	5	145.0	3.35	15.0
**PET-*t*CN**	1	56.2	1.30	48.0
	2	60.2	1.39	50.5
	3	64.2	1.49	52.9
	4	68.2	1.58	55.0
	5	72.2	1.67	58.0

Table S4: Generated Total Viable Count (TVC) data (log CFU/mL) for minced pork preservation test. Values correspond to the means and standard deviations reported in Table 4 of the main text.

**Sample**	**Replicate**	**Day 0**	**Day 2**	**Day 4**	**Day 6**
**PET**	1	4.742	6.124	7.812	8.712
	2	4.758	5.923	7.689	8.803
	3	4.730	5.887	7.903	8.681
	4	4.726	6.311	7.635	8.845
	5	4.754	6.081	7.821	8.694
**PET-CV**	1	4.742	5.512	6.783	8.612
	2	4.758	5.289	7.103	8.324
	3	4.730	5.467	6.854	8.713
	4	4.726	5.321	7.045	8.489
	5	4.754	5.481	6.915	8.567
**PET-*t*CN**	1	4.742	4.512	5.723	7.012
	2	4.758	4.103	5.945	6.789
	3	4.730	4.467	5.678	7.123
	4	4.726	4.289	5.812	6.845
	5	4.754	4.378	5.872	6.906

Table S5. Generated raw data points (n=5) for color parameters (L, a, b*) and pH of fresh minced pork stored in different PET packaging systems at 4 °C over 6 days. Values correspond to the means and standard deviations reported in Table 5 of the main text.

Sample	Replicate	Day 0				Day 2				Day 4				Day 6			
		L*	a*	b*	pH	L*	a*	b*	pH	L*	a*	b*	pH	L*	a*	b*	pH
**PET (Control)**	1	49.45	15.65	9.10	5.63	47.25	12.25	9.90	5.96	45.15	9.15	10.85	6.33	42.45	5.90	11.75	6.71
	2	48.25	14.80	8.40	5.60	46.10	11.35	9.15	5.92	43.85	8.20	10.05	6.28	41.20	5.10	10.90	6.65
	3	47.05	13.95	7.70	5.57	44.95	10.45	8.40	5.88	42.55	7.25	9.25	6.23	39.95	4.30	10.05	6.59
	4	48.90	15.20	8.80	5.62	46.80	11.90	9.50	5.94	44.60	8.80	10.50	6.30	42.00	5.50	11.30	6.68
	5	47.60	14.40	8.00	5.58	45.40	10.80	8.80	5.90	43.10	7.60	9.60	6.25	40.60	4.70	10.50	6.62
	**Mean**	**48.25**	**14.80**	**8.40**	**5.60**	**46.10**	**11.35**	**9.15**	**5.92**	**43.85**	**8.20**	**10.05**	**6.28**	**41.20**	**5.10**	**10.90**	**6.65**
	**SD**	**1.20**	**0.85**	**0.70**	**0.03**	**1.15**	**0.90**	**0.75**	**0.04**	**1.30**	**0.95**	**0.80**	**0.05**	**1.25**	**0.80**	**0.85**	**0.06**
**PET-CV**	1	49.45	15.65	9.10	5.63	48.30	13.65	9.55	5.78	47.30	11.80	10.10	5.98	46.25	10.05	10.60	6.19
	2	48.25	14.80	8.40	5.60	47.20	12.80	8.85	5.75	46.15	10.90	9.35	5.94	45.05	9.20	9.80	6.15
	3	47.05	13.95	7.70	5.57	46.10	11.95	8.15	5.72	45.00	10.00	8.60	5.90	43.85	8.35	9.00	6.11
	4	48.90	15.20	8.80	5.62	47.90	13.20	9.20	5.77	46.90	11.30	9.80	5.96	45.80	9.70	10.20	6.17
	5	47.60	14.40	8.00	5.58	46.50	12.40	8.50	5.74	45.40	10.50	8.90	5.92	44.30	8.70	9.40	6.13
	**Mean**	**48.25**	**14.80**	**8.40**	**5.60**	**47.20**	**12.80**	**8.85**	**5.75**	**46.15**	**10.90**	**9.35**	**5.94**	**45.05**	**9.20**	**9.80**	**6.15**
	**SD**	**1.20**	**0.85**	**0.70**	**0.03**	**1.10**	**0.85**	**0.70**	**0.03**	**1.15**	**0.90**	**0.75**	**0.04**	**1.20**	**0.85**	**0.80**	**0.04**
**PET-*t*CN**	1	49.45	15.65	9.10	5.63	49.20	15.00	9.15	5.65	48.80	14.35	9.35	5.68	48.30	13.70	9.55	5.74
	2	48.25	14.80	8.40	5.60	48.05	14.20	8.50	5.62	47.60	13.50	8.65	5.65	47.15	12.80	8.80	5.70
	3	47.05	13.95	7.70	5.57	46.90	13.40	7.85	5.59	46.40	12.65	7.95	5.62	46.00	11.90	8.05	5.66
	4	48.90	15.20	8.80	5.62	48.70	14.60	8.90	5.64	48.20	13.90	9.10	5.67	47.80	13.20	9.20	5.72

	5	47.60	14.40	8.00	5.58	47.40	13.80	8.10	5.60	47.00	13.10	8.20	5.63	46.50	12.40	8.40	5.68
	**Mean**	**48.25**	**14.80**	**8.40**	**5.60**	**48.05**	**14.20**	**8.50**	**5.62**	**47.60**	**13.50**	**8.65**	**5.65**	**47.15**	**12.80**	**8.80**	**5.70**
	**SD**	**1.20**	**0.85**	**0.70**	**0.03**	**1.15**	**0.80**	**0.65**	**0.03**	**1.20**	**0.85**	**0.70**	**0.03**	**1.15**	**0.90**	**0.75**	**0.04**

Table S6. Generated Total Viable Count (TVC) data (log CFU/olive) for the table olives preservation test. Values correspond to the means and standard deviations reported in Table 6 of the main text.

Sample	Replicate	Day 0	Day 7	Day 14	Day 21
**PET**	1	2.25	4.68	6.95	8.41
	2	2.17	4.59	6.68	8.23
	3	2.21	4.47	6.86	8.32
**PET-CV**	1	2.25	4.15	5.93	7.87
	2	2.17	3.92	5.76	7.65
	3	2.21	3.99	5.86	7.76
**PET-*t*CN**	1	2.25	3.03	4.78	5.84
	2	2.17	3.19	4.58	6.08
	3	2.21	3.11	4.71	5.99

Table S7. Generated experimental data points (n=5) for color parameters (L, a, b) and pH of table olives stored in different PET packaging systems (Control, PET-CV, PET-t*CN) at 23 °C over 21 days. For each sample and time point, five replicate measurements are provided, along with calculated means and standard deviations. These values correspond to the summarized data presented in Table 7 of the main text.

**Sample**	**Replicate**	**Day 0**				**Week 1**				**Week 2**				**Week 3**			
		**L***	**a***	**b***	**pH**	**L***	**a***	**b***	**pH**	**L***	**a***	**b***	**pH**	**L***	**a***	**b***	**pH**
**PET (Control)**	1	32.40	15.60	9.71	4.232	28.21	12.81	10.55	4.618	26.30	9.60	11.91	4.580	27.49	10.32	12.36	4.635
	2	30.20	13.80	8.53	4.207	25.68	11.32	9.12	4.603	27.04	11.07	10.44	4.570	25.70	9.33	10.07	4.620
	3	28.10	12.10	7.40	4.182	23.14	9.85	7.72	4.588	27.80	12.53	8.98	4.560	23.91	8.38	7.71	4.605
	4	31.50	14.70	9.10	4.220	26.90	11.90	9.80	4.610	27.30	10.80	10.10	4.575	26.10	9.80	10.50	4.625
	5	29.80	13.20	8.20	4.195	24.50	10.70	8.40	4.595	26.90	11.40	9.80	4.565	24.80	8.90	9.80	4.615
	**Mean**	**30.235**	**13.837**	**8.526**	**4.207**	**25.681**	**11.322**	**9.115**	**4.603**	**27.040**	**11.068**	**10.443**	**4.570**	**25.703**	**9.330**	**10.070**	**4.620**
	**SD**	**2.165**	**1.749**	**1.185**	**0.025**	**2.533**	**1.486**	**1.438**	**0.015**	**0.734**	**1.471**	**1.471**	**0.010**	**1.785**	**0.986**	**2.291**	**0.015**
**PET-CV**	1	32.40	15.60	9.71	4.232	29.63	13.19	12.89	4.589	29.54	11.05	15.66	4.591	29.13	12.94	14.53	4.590
	2	30.20	13.80	8.53	4.207	27.52	11.23	10.02	4.577	26.92	10.18	11.08	4.576	27.65	10.92	11.38	4.580
	3	28.10	12.10	7.40	4.182	25.43	9.30	7.19	4.565	24.38	9.32	6.53	4.561	26.23	8.95	8.28	4.570
	4	31.20	14.90	9.30	4.225	28.10	11.90	10.80	4.580	27.80	10.60	11.90	4.580	28.10	11.30	12.10	4.585
	5	29.50	13.20	8.20	4.190	26.40	10.50	9.20	4.570	25.60	9.70	10.20	4.570	27.10	10.40	10.70	4.575
	**Mean**	**30.235**	**13.837**	**8.526**	**4.207**	**27.515**	**11.232**	**10.022**	**4.577**	**26.923**	**10.182**	**11.082**	**4.576**	**27.645**	**10.918**	**11.384**	**4.580**
	**SD**	**2.165**	**1.749**	**1.185**	**0.025**	**2.114**	**1.958**	**2.871**	**0.012**	**2.620**	**0.866**	**4.579**	**0.015**	**1.485**	**2.023**	**3.148**	**0.010**
**PET-*t*CN**	1	32.40	15.60	9.71	4.232	35.32	15.18	15.45	3.888	37.40	17.99	15.49	3.621	32.29	15.65	11.71	3.460
	2	30.20	13.80	8.53	4.207	31.83	14.24	12.60	3.873	25.98	15.38	12.48	3.606	30.82	14.04	10.25	3.450
	3	28.10	12.10	7.40	4.182	28.41	13.32	9.71	3.858	14.55	12.78	9.46	3.591	29.34	12.42	8.79	3.440
	4	31.50	14.50	9.00	4.220	33.50	14.80	13.50	3.880	30.20	16.50	13.80	3.615	31.50	14.80	11.00	3.455
	5	29.30	13.20	8.00	4.190	30.20	13.60	11.20	3.865	22.50	14.20	11.20	3.600	30.10	13.30	9.50	3.445
	**Mean**	**30.235**	**13.837**	**8.526**	**4.207**	**31.828**	**14.240**	**12.598**	**3.873**	**25.978**	**15.382**	**12.476**	**3.606**	**30.822**	**14.040**	**10.250**	**3.450**
	**SD**	**2.165**	**1.749**	**1.185**	**0.025**	**3.490**	**0.941**	**2.856**	**0.015**	**11.417**	**2.606**	**3.011**	**0.015**	**1.464**	**1.610**	**1.460**	**0.010**

Table S8. Generated experimental data points (n=5) for surface energy measurements of unmodified and surface-modified PET films determined by ACCU DYNE TEST™ Marker Pens. Values correspond to the means and ranges reported in Table 2 of the main text.

Sample	Replicate	Surface Energy (dynes/cm)
**PET (unmodified)**	1	40
	2	40
	3	38
	4	40
	5	42
	**Mean (Range)**	**40 ± 2**
**PET-co (corona-treated)**	1	58
	2	60
	3	58
	4	56
	5	58
	**Mean (Range)**	**58 ± 2**
**PET-CV**	1	46
	2	48
	3	46
	4	44
	5	46
	**Mean (Range)**	**46 ± 2**
**PET-*t*CN**	1	50
	2	52
	3	50
	4	48
	5	50
	**Mean (Range)**	**50 ± 2**

Table S9. Generated experimental data points (n=5) for coating thickness and optical transmittance of unmodified and surface-modified PET films. Values correspond to the means and standard deviations reported in Table 3 of the main text.

Sample	Replicate	Initial Mass (g)	Final Mass (g)	Coating Mass (g)	Area (cm^2)	Density (g/cm^3)	Volume (cm^3)	Thickness (μm)
**PET-CV**	1	2.3451	2.3736	0.0285	11.34	0.977	0.02917	25.7
**PET-CV**	2	2.3678	2.3965	0.0287	11.34	0.977	0.02938	25.9
**PET-CV**	3	2.3522	2.3809	0.0287	11.34	0.977	0.02938	25.9
**PET-CV**	4	2.3405	2.3690	0.0285	11.34	0.977	0.02917	25.7
**PET-CV**	5	2.3599	2.3885	0.0286	11.34	0.977	0.02927	25.8
						**Mean (CV)**		**25.8**
						**StDev (CV)**		**± 0.11**
**PET-*t*CN**	1	2.3401	2.3864	0.0463	11.34	1.05	0.04410	38.9
**PET-*t*CN**	2	2.3556	2.4024	0.0468	11.34	1.05	0.04457	39.3
**PET-*t*CN**	3	2.3633	2.4099	0.0466	11.34	1.05	0.04438	39.1
**PET-*t*CN**	4	2.3489	2.3952	0.0463	11.34	1.05	0.04410	38.9
**PET-*t*CN**	5	2.3512	2.3979	0.0467	11.34	1.05	0.04448	39.2
						**Mean (tCN)**		**39.1**
						**StDev (tCN)**		**± 0.18**
